# The effects of hydrocortisone and yohimbine on human behavior in approach-avoidance conflicts

**DOI:** 10.1007/s00213-023-06396-6

**Published:** 2023-06-14

**Authors:** Kim Fricke, Nina Alexander, Thomas Jacobsen, Henriette Krug, Kai Wehkamp, Susanne Vogel

**Affiliations:** 1 Department of Psychology, Medical School Hamburg, Am Kaiserkai 1, 20457 Hamburg, Germany; 2 ICAN Institute for Cognitive and Affective Neuroscience, Medical School Hamburg, Am Kaiserkai 1, 20457 Hamburg, Germany; 3 Department of Psychiatry and Psychotherapy, Philipps University Marburg, Rudolf-Bultmann-Str. 8, 35039 Marburg, Germany; 4 Experimental Psychology Unit, Helmut-Schmidt-University/University of the Federal Armed Forces Hamburg, Holstenhofweg 85, 22043 Hamburg, Germany; 5 Faculty of Health Sciences, Medical School Hamburg, Am Kaiserkai 1, 20457 Hamburg, Germany

**Keywords:** Humans, Yohimbine, Hydrocortisone, Noradrenaline, Cortisol, Testosterone, Estrogen, Avoidance behavior, Approach behavior

## Abstract

**Rationale:**

Balancing approach of positive and avoidance of negative stimuli is essential when faced with approach-avoidance conflicts, e.g., situations with both positive and negative outcomes. This balance is disturbed in several mental disorders, e.g., excessive avoidance in anxiety disorders, and heightened approach in substance use disorders. Since stress is assumed to impact these disorders’ etiology and maintenance, it seems crucial to understand how stress influences behavior in approach-avoidance conflicts. Indeed, some studies suggested altered approach-avoidance behavior under acute stress, but the mechanism underlying these effects is unknown.

**Objectives:**

Investigate how the pharmacological manipulation of major stress mediators (cortisol and noradrenaline) influences task-based approach-avoidance conflict behavior in healthy individuals.

**Methods:**

Ninety-six participants (48 women, 48 men) received either 20mg hydrocortisone, 20mg yohimbine, both, or placebo before performing a task targeting foraging under predation in a fully crossed double-blind between-subject design. Moreover, we investigated effects of gender and endogenous testosterone and estradiol levels on approach-avoidance behavior.

**Results:**

While biological stress markers (cortisol concentration, alpha amylase activity) indicated successful pharmacological manipulation, behavior in approach-avoidance conflicts was not affected as expected. Although yohimbine administration affected risky foraging latency under predation, we found no main effect of hydrocortisone or their interaction on behavior. In contrast, we found gender differences for almost all behavioral outcome measures, which might be explained by differences in endogenous testosterone levels.

**Conclusions:**

The investigated major stress mediators were not sufficient to imitate previously shown stress effects on approach-avoidance conflict behavior. We discuss potential reasons for our findings and implications for future research.

**Supplementary Information:**

The online version contains supplementary material available at 10.1007/s00213-023-06396-6.

## Introduction

Approach and avoidance are highly conserved behaviors across both species and time. Stimuli of positive valence are approached, while negative stimuli are avoided via generalized goal-oriented systems sensitive to reward and punishment (Gray 1975). These systems also mediate conflict resolution in situations in which conflicting stimuli (or features) are present, for example, both reward and punishment. Importantly, these systems are sensitive to the perceived distance between oneself and either reward or punishment. Rewards elicit approach even at long distances, while avoidance of punishment outweighs approach motivations when close to the punishing stimulus. Distance also influences the behavioral response itself, for example, when faced with threat (fight, flight, or freeze response; Blanchard and Blanchard 1988; McNaughton et al. 2016).

Approach-avoidance behaviors are imbalanced in many mental disorders, e.g., excessive avoidance in anxiety disorders or disproportionate approach in pathological aggression and substance use disorders (Carver and Harmon-Jones 2009; Wiers et al. 2014; World Health Organization 1992). Since stress is implied in both onset and maintenance of mental disorders (e.g., Koob et al. 2014; Shin and Liberzon 2010), some studies have investigated the impact of acute stress on approach-avoidance behaviors, utilizing different paradigms and resulting in equivocal effects (see Fricke and Vogel 2020 for a recent overview). These equivocal effects could be due to a more nuanced effect of stress on approach-avoidance behavior as a recent study suggested (Vogel and Schwabe 2019). There, healthy participants underwent a psychosocial stressor before performing the approach-avoidance conflict task (AACT; Bach et al. 2014). In the AACT, foraging for monetary rewards under threat is encouraged, creating an ambiguous situation with conflicting approach and avoidance motivations. While stress did not have strong general effects on risky foraging, it increased the importance of threat distance: stressed participants displayed faster escape responses when threat was close (active avoidance) compared to further away (passive avoidance, i.e., inhibition of behavior when faced with distant threats). Additionally, stress further led to differences in approach-avoidance behavior based on the participants’ individual trait anxiety and aggression, seemingly abolishing differences in trait anxiety, while amplifying approach behaviors in more physically aggressive individuals. Stress was therefore shown to override or exacerbate the effects of personality traits for trait anxiety and aggression, respectively, suggesting that differences in approach-avoidance behavior may be closely linked to both acute levels of stress and more stable personality traits. However, the mechanism of *how* stress affects approach-avoidance behavior is unclear.

Although stress comes with a multitude of physiological changes, often two major subsystems of the stress response are investigated with regard to cognitive effects, namely the hypothalamic-pituitary-adrenal (HPA) axis and the sympathetic nervous system (SNS; Ulrich-Lai and Herman 2009). While the combined roles of cortisol as major end product of the HPA axis and noradrenaline (NA) as main neurotransmitter of the SNS in balancing approach and avoidance have not been investigated as of yet, their interaction has been clearly demonstrated as the mechanism underlying acute stress effects in other cognitive domains such as memory consolidation (Barsegyan et al. 2010; Barsegyan et al. 2019; Quirarte et al. 1997; Roozendaal et al. 2006b), instrumental learning (Schwabe et al. 2010, 2012), and fear conditioning (Roozendaal et al. 2006a). In the case of approach-avoidance behavior, few studies have investigated either of the systems, but no study investigated their interaction. Endogenous and pharmacologically administered cortisol has been investigated in a few task-based approach-avoidance studies, which were highly varied in their designs, participants, and results and suggested interactions of cortisol effects with interindividual differences (Dapprich et al. 2021; Roelofs et al. 2009; Van Peer et al. 2007). For NA, we found only one study, which indicated no effect of noradrenergic stimulation on approach-avoidance behavior (Deuter et al. 2021). Importantly, these studies did not include a manipulation of threat distance which has a critical impact on approach-avoidance behavior.

To investigate the effects of cortisol, NA, and their interaction on the balance of approach-avoidance conflict behavior, 96 healthy participants received a double-blinded pharmacological intervention to increase their cortisol concentration (20mg hydrocortisone), activity of NA (20mg yohimbine), both, or neither. Afterwards, participants performed the AACT in which they foraged for tokens (approach motivation) under probabilistic threat of virtual predators (avoidance motivation). Importantly, this task included a manipulation of initial threat distance.

We hypothesized that, in line with research from other cognitive domains, the combination of both cortisol and NA would mimic previously shown stress effects on approach-avoidance behavior (Vogel and Schwabe 2019). Therefore, the combined administration of cortisol and NA should amplify the importance of threat distance for avoidance behaviors. As the study by Vogel and Schwabe (2019) suggested more nuanced stress effects based on interindividual differences, we hypothesized our intervention to increase approach behavior in more trait aggressive individuals and abolish effects of trait anxiety differences. We further expected more approach behavior in trait aggressive and sensation-seeking individuals and more avoidance behavior in trait anxious participants, independent of drug condition. Finally, we expected men to perform better than women based on previous AACT studies (Bach et al. 2020; Vogel and Schwabe 2019). Due to striking gender differences, we exploratively investigated effects of basal testosterone and estradiol levels on task-based approach-avoidance behavior as they have not been extensively investigated (Fricke and Vogel 2020).

## Experimental procedures

### Participants

Our recruitment strategy as well as detailed exclusion and inclusion criteria, also in respect to factors affecting the HPA axis or the noradrenergic nervous system, can be found in Online Resource 1. Ninety-six healthy individuals (48 self-identified men, 48 self-identified women, age: 18–35 years; mean: 24.69; SD: 4.47) completed the experiment. Two noncompliant participants were excluded, leading to a total sample size of 94 participants. The target sample size of 96 was supported by an a-priori power analysis, allowing the discovery of medium-sized effects at an alpha error probability of .05 and a power of 80% for repeated measures ANOVAs with between participant variables resulting in four groups (G*Power 3.1.9.7; Faul et al. 2007). Participants provided written informed consent and received monetary compensation (30 Euro; or 5 Euro and partial course credit) for participation. The study was approved by the local ethics committee (Ethik-Kommission der Ärztekammer Hamburg; PV 5310).

To determine how cortisol, NA, and their interaction affect the balance of approach- and avoidance-behavior, we employed a fully crossed double-blind between-subject design. Participants were pseudo-randomly assigned to four groups, while balancing for gender, and received (1) 20 mg hydrocortisone and placebo (*n*=23), (2) 20 mg yohimbine and placebo (*n*=23), (3) 20 mg hydrocortisone and 20 mg yohimbine (*n*=24), or (4) placebo (*n*=24) via three identical-looking pills containing 20 mg hydrocortisone, 10 mg yohimbine, or placebo.

### Experimental procedures

Participants were tested between 12:20 and 18:45 to control for the diurnal rhythm of cortisol. They were instructed to arrive well-rested, limit themselves to light physical exercise on the day of the experiment, and avoid the use of alcohol and other psychoactive substances starting the day prior to the experiment. Participants were asked to have a light meal roughly 2 h before the experiment and avoid food and drink intake (except water) in the 30 min leading up to the experiment.

After arrival, participants answered questionnaires assessing current mood (MDBF; Steyer et al. 1994, and three visual analog scales (VAS; anxious, upset, stressed)), trait anxiety (STAI-T; Laux et al. 1981), chronic stress (TICS; Schulz et al. 2004; not reported here), trait aggression (DAF; Werner and von Collani 2014), and sensation seeking (SSS-V; Beauducel et al. 2003). This was followed by a baseline measurement (T1) of vital signs (heart rate, diastolic, and systolic blood pressure) and saliva sampling (cortisol concentration, alpha amylase activity as marker of noradrenergic activity, testosterone and estradiol concentrations). Detailed descriptions of vital sign and saliva sampling methods can be found in Online Resource 2.

After baseline measurements, participants performed a risk-taking task, the Balloon Analogue Risk Task (BART; Lejuez et al. 2002; not reported here), and received the assigned pharmacological intervention orally. Afterwards, saliva and vital signs were taken every 15 min for a total of three times (T2-4), during which participants were allowed to read. Another assessment of current mood and a slightly altered BART followed. This protocol was in line with previous studies for both the dosages and the 45-min waiting period before the onset of the first task (Schwabe et al. 2010, 2012; Schwabe and Wolf 2013).

Approximately 65 min after medication intake, participants began the AACT (average duration: 42 min), followed by final saliva sample, vital signs, and mood assessments (T5). Finally, participants indicated which pharmacological intervention they believed to have received and were debriefed about study procedures. The experiment lasted about 2 h and 40 min.

### Approach-avoidance conflict task and outcome parameters

**Fig. 1:**
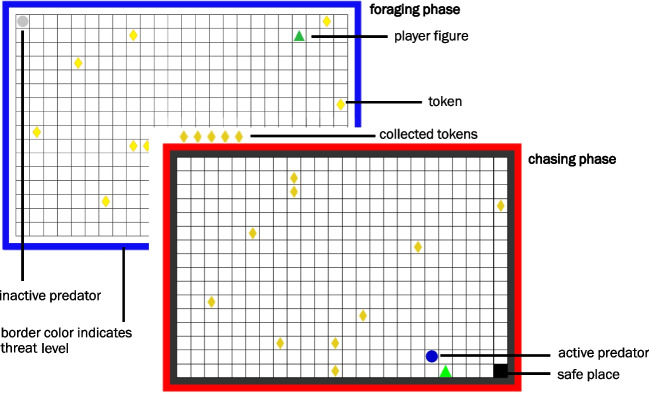
Approach-avoidance conflict task, adapted from Bach et al. (2014). Participants were tasked to collect as many of the tokens placed at random as they could, while avoiding capture by a predator. Trials (160 in 4 blocks) varied in time between 6 and 15 s after which the predator woke up in 20% (low threat; 50% of trials) or 60% of trials (high threat) and chased the participant for 3.5 s, if not caught. The predator is at minimum 2.5 times faster than the player. At trial start, the player figure is placed next to the predator or in the safe place (where the predator could not catch them) in 50% of trials each. In the foraging phase, the border color indicates which threat level is present in the current trial, while on predator wake-up, the color changes to red. For more details, see Online Resource 3. The figure has been re-used with permission from Fricke and Vogel (2020)

To assess approach-avoidance behavior, we employed an adapted version of the AACT (programmed in Python 3.2.5 using Pygame 1.9.2 and made available under osf.io/d69pr; see Vogel and Schwabe 2019), originally developed by Bach et al. (2014); see Fig. 1). In each of the 160 trials (evenly divided into four blocks), participants foraged for tokens under threat of predation (high vs. low threat condition based on the probability of predator waking up) and started either close to the predator or far away in a predator-safe space to manipulate threat distance. After the AACT, participants were asked to estimate the wake-up probabilities for both predators. For a detailed description of the AACT, see Online Resource 3.

Due to the nature of the task, e.g., the ability to move freely and the intertwined goals of approach and avoidance, many variables can be considered outcome variables (Bach et al. 2020). Here, we focused on three previously established summary measures (Bach et al. 2018; Korn et al. 2017; Vogel and Schwabe 2019): foraging latency (initial time to first button press) can be informative for the initial decision process within each trial, the sum of retained tokens (sum of tokens collected in all trials unless the participants were caught) as overall performance measure, and failed avoidance of threat (the rate at which participants were caught) as an additional performance measure, especially regarding risk proneness during the AACT.

### Statistical analysis

To assess potential group differences in control variables (age, BMI) and personality traits, ANOVAs with the between-subject factor group were employed. Potential biases in sample composition over time due to the onset and development of the COVID-19 pandemic were explored in Online Resource 4. Successful blinding was assessed by testing participants’ ability to correctly identify whether they had received an active treatment, and if so, which treatment in particular, with chi-square and Fischer’s exact test. To test whether the administration of hydrocortisone and yohimbine had the expected effects on physiological measures (heart rate, blood pressure, cortisol concentration, alpha amylase activity) and affected subjective mood (MDBF, VAS), we conducted mixed-design ANOVAs with the within-subject factor time and the between-subject factors hydrocortisone (vs. placebo) and yohimbine (vs. placebo). We report main and interaction effects involving either or both drugs. Due to strong variability of data for both cortisol concentration and alpha amylase activity, a 10% winsorizing of the data was employed as outlier correction. To correct for multiple comparisons, we applied Bonferroni-Holm corrections based on outcome variables for five physiological and six subjective measures, respectively.

Regarding the AACT, we first assessed whether drug administration affected explicit task knowledge (i.e., estimated wake-up probabilities of predators) via a mixed-design ANOVA with the within-subject factor threat level (high vs. low) and the between-subject factors hydrocortisone, yohimbine, and gender. Then, three previously established summary measures over all trials, i.e., foraging latency, sum of retained tokens, and failed avoidance of threat, were investigated. We conducted mixed-design ANOVAs with the within-subject factors initial threat distance (long vs. short), threat level and block (1 to 4), and the between-subject factors hydrocortisone, yohimbine, and gender (Bonferroni-Holm corrected for three outcome variables). For analyses on failed avoidance of threat, participants were excluded if their data contained empty cells (remaining *n*=59; placebo: *n*=16, hydrocortisone: *n*=14, yohimbine: *n*=15, hydrocortisone and yohimbine: *n*=14) due to a programming error (task blocks missing combinations of threat level × threat distance × threat wake-up for individual participants). An exploratory analysis omitting block as within-subject factor (thus including all participants) was conducted to assure that potential drug effects would not be lost due to fewer participants in the initial analysis.

To investigate the influence of personality traits (trait anxiety by STAI-T total score (Laux et al. 1981), sensation seeking by SSSV total score (Beauducel et al. 2003) and aspects of trait aggression by four subscales of the DAF, namely physical aggression, verbal aggression, anger, and mistrust (Werner and von Collani 2014)) on AACT performance, we focused on the sum of tokens retained. This decision allows for comparability with prior work (Vogel and Schwabe 2019) which assessed the relationship of STAI-T and DAF subscales with AACT performance under stressful and non-stressful conditions. In addition, a recent study revealed token retention as one of the most reliable task parameters, supporting our decision for this variable (Bach et al. 2020). First, we correlated each personality trait with the sum of tokens retained (Bonferroni-Holm corrected for six comparisons). To understand the influences of the pharmacological interventions, we conducted hierarchical linear regressions with introduction of the following mean-centered variables in blockwise fashion: (1) The control variables gender, age, and average movement speed during the AACT, followed by (2) the interventions (hydrocortisone, yohimbine) as well as their interaction, (3) the personality trait measures detailed above, and (4) the interactions of personality trait measures with the interventions.

To explore whether basal endogenous testosterone or estradiol affected approach-avoidance behavior, we conducted exploratory correlations between sex hormone concentrations and our summary outcome measures, and hierarchical regressions for these outcome measures with blockwise introduction of (1) the control variable age and (2) either testosterone or estradiol once for all participants and additionally separated by gender. ANOVAs were used to assess whether the full model explained variance significantly better than the respective control model. Participants were excluded for the respective analyses, if their testosterone/estradiol levels differed three or more standard deviations from the mean.

To enhance comparability with previous findings (e.g., Bach et al. 2014; Bach et al. 2018; Bach et al. 2020; Korn et al. 2017), we also reanalyzed our primary hypotheses with ANOVAs for six previously described outcome variables over time-in-trial (e.g., time spent in safe space; token collection rate) as well as for outcome parameters identified as test-retest reliable over 11–32 months. For the outcome parameters chosen for their test-retest reliability, we included personality trait analyses similar to the one above regarding the sum of tokens (see Online Resource 5). As suggested by a reviewer, we also investigated in how far threat overestimation related to different AACT outcome measures (see Online Resource 6).

For significant findings in the ANOVAs detailed above, the appropriate follow-up tests, including ANOVAs and *t*-tests, were conducted. When sphericity was violated, we employed Greenhouse-Geisser correction. Post hoc Bonferroni-Holm corrections for multiple testing were based on the number of separate post hoc ANOVAs or *t*-tests per analysis. All reported *p*-values are two-tailed. All analyses were conducted in R (Version 4.2.2) and can be found at osf.io/d69pr.

## Results

The four experimental groups did not differ in age (*p*=.517), BMI (*p*=.423), trait anxiety (*p*=.584), trait aggression (all scales *p*≥.411), or sensation seeking (*p*=.221, Online Resource Table S1). Participants were successfully blinded to their treatment as they could not differentiate active medication from placebo intake (*X*^2^_3,N=94_=2.312, *p*=.510), or guess the exact combination of medication they had been given (*p*=.227).

### Hydrocortisone and yohimbine administrations affect biological stress markers, but not self-reported mood

**Fig. 2:**
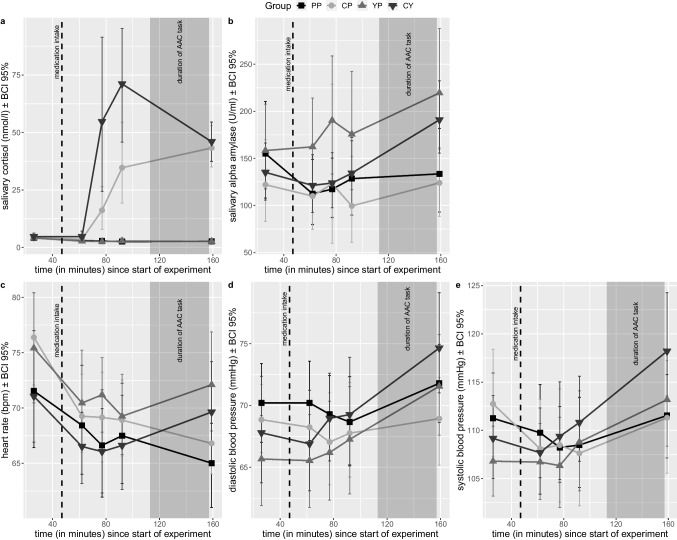
Measures of (**a**) salivary cortisol concentration, (**b**) salivary alpha amylase activity, (**c**) heart rate, (**d**) diastolic blood pressure, and (**e**) systolic blood pressure over time. Dotted line indicates time of medication intake. Grey overlay indicates duration of the approach-avoidance conflict task. Error bars represent bootstrapped 95% confidence intervals (BCI). Groups: placebo (PP), hydrocortisone (CP), yohimbine (YP), hydrocortisone and yohimbine (CY)

As expected, hydrocortisone and yohimbine intake led to pronounced increases in cortisol concentration and activation of the noradrenergic system, respectively. Salivary cortisol concentration changed over time dependent on hydrocortisone administration (*F*_1.78,149.67_=18.832, *p*<.001, η^2^G=.126; see Fig. 2a). Post hoc ANOVAs showed significant increases after taking hydrocortisone compared to not taking hydrocortisone (T3: 36.32 vs. 2.69 nmol/l, *F*_1,84_=11.231, *p*=.003, η^2^G=.118; T4: 53.73 vs. 2.69 nmol/l, *F*_1,84_=36.390, *p*<.001, η^2^G=.302; T5: 44.68 vs. 2.62 nmol/l, *F*_1,84_=158.749, *p*<.001, η^2^G=.654). After winsorization, a time-dependent hydrocortisone-yohimbine interaction (*F*_1.86, 156.04_=5.181, *p*=.040, η^2^G=.039) suggested that yohimbine intake further increased salivary cortisol concentration in hydrocortisone-taking participants. However, post hoc ANOVAs did not indicate additional significant differences at any time point. Salivary alpha amylase activity did not differ between treatment groups (see Fig. 2b). After winsorization, we found the expected interaction of yohimbine and time (*F*_2.79,229.19_=3.238, *p*=.026, η^2^G=.012), resulting from higher alpha amylase activity in groups with yohimbine intake at T3 (raw values: 155.60 vs. 120.04 U/ml; *F*_1,82_=7.146, *p*=.036, η^2^G=.080) and T5 (204.61 vs. 128.99 U/ml; *F*_1,82_=10.700, *p*=.010, η^2^G=.115) compared to the other groups.

Vital signs changed over time dependent on the intake of yohimbine (heart rate: *F*_3.03,272.68_=7.889, *p*<.001, η^2^G=.010; diastolic blood pressure: *F*_3.35,301.47_=6.583, *p*<.001, η^2^G=.013); systolic: *F*_3.18,286.11_=9.387, *p*<.001, η^2^G=.014; see Fig. 2c–e), but not hydrocortisone. However, post hoc ANOVAs indicated no specific time point for those significant differences. Subjective mood was not affected by hydrocortisone or yohimbine intake, supporting successful blinding of our pharmacological intervention (see Online Resource Table S2).

### Participants overestimate low threat condition in the approach-avoidance conflict task

Participants differentiated between high- and low-threat predators (mean estimated wake-up probability 38.2% vs. 61.6%, *F*_1,86_=34.882, *p*<.001, η^2^G=.218), but overestimated the low-threat predator by around 20%. Threat level ratings were further affected by hydrocortisone administration (*F*_1,86_=4.734, *p*=.032, η^2^G=.036), but post hoc tests revealed no significant effects. In general, women overestimated threat more than men (mean overestimation of wake-up probability 12.0% vs. 7.7%, *F*_1,86_=5.454, *p*=.022, η^2^G=.019, see Online Resource Figure S1). For additional analyses regarding influences of threat overestimation on summary outcome variables, see Online Resource 6. No other effects of gender, drug treatment, or their interaction reached significance.

### No hypothesized effects of hydrocortisone and yohimbine on behavior

**Fig. 3:**
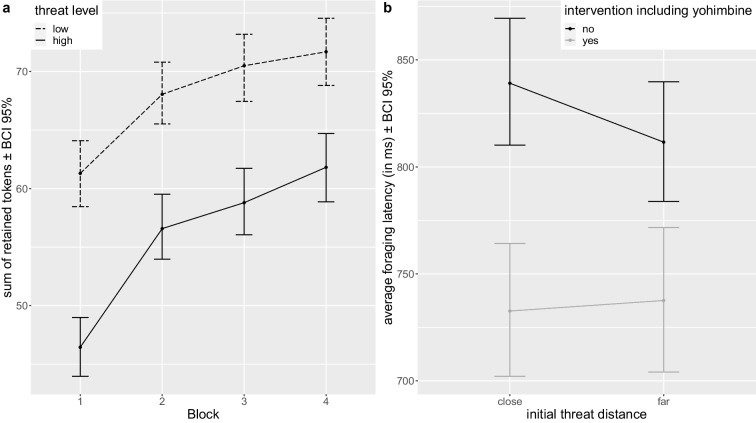
**a** Differences in initial latency to avoid/approach based on the initial threat distance and yohimbine administration (independent of whether hydrocortisone was administered). **b** Averaged retained tokens over blocks for the low and high threat level conditions. Error bars represent bootstrapped 95% confidence intervals (BCI)

In general, participants improved their performance over time. They were caught less frequently after the first block (*F*_3,153_=8.973, *p*<.001, η^2^G=.025, block 1 53.1% vs. block 2 40.4%, *t*_235_=5.03, *p*<.001, Cohen’s *d*=.328) and retained more tokens over blocks (*F*_2.61,224.82_=115.242, *p*<.001, η^2^G=.071), which interacted with threat level (*F*_3,258_=4.549, *p*=.012, η^2^G=.003, see Fig. 3a). Post hoc tests showed that participants retained more tokens in the second compared to the first block for both threat levels (low threat: *t*_187_=−4.88, *p*<.001, Cohen’s *d*=−.356; high threat: *t*_187_=−7.14, *p*<.001, Cohen’s *d*=−.521). In addition, participants retained more tokens when threat level was low (*F*_1,86_=246.457, *p*<.001, η^2^G=.102), but were also caught more often if the predator awoke (mean catch rate 47.4% vs. 41.7%, *F*_1,51_=7.737, *p*=.016, η^2^G=.010). When initial threat distance was long (compared to short), participants retained less tokens (sum of retained tokens 59 vs. 64, *F*_1,86_=75.760, *p*<.001, η^2^G=.024) suggestive of behavioral inhibition.

**Fig. 4:**
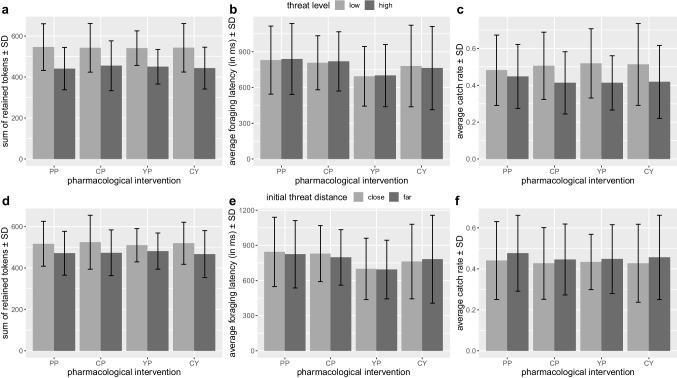
Averaged main outcome measures (left (**a**, **d**): sum of retained tokens, middle (**b**, **e**): average foraging latency, and right (**c**, **f**): average catch rate) per experimental group displayed either per threat level (top panel) or initial threat distance (bottom panel). Error bars represent one standard deviation. Groups: placebo (PP), hydrocortisone (CP), yohimbine (YP), hydrocortisone and yohimbine (CY)

Against our hypothesis, we did not find any hydrocortisone-yohimbine interaction or hydrocortisone main effect on overall task performance (see Fig. 4a–f). For yohimbine, an interaction with threat distance was found for foraging latency (*F*_1,86_=6.494, *p*=.039, η^2^G=.001), indicating that solely participants without yohimbine administration approached foraging faster when initial threat distance was long as compared to short (mean latency 812 vs. 839 ms, *F*_1,43_=13.902, *p*=.001, η^2^G=.003, see Fig. 3b). Analyses of pharmacological effects on further AACT outcome variables (Online Resource 5) likewise did not reveal the hypothesized effects of hydrocortisone, yohimbine, or their interaction on approach-avoidance conflict behavior. Our hypotheses regarding the pharmacological intervention were thus not confirmed.

### Correlations and hierarchical regressions show no significant relationships between AACT performance and personality traits, pharmacological interventions, or their interactions

**Fig. 5:**
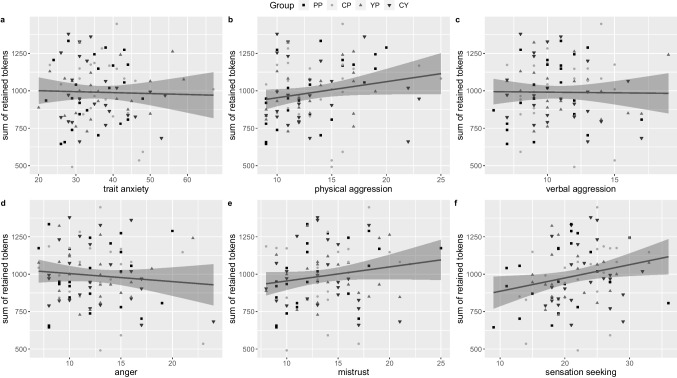
Correlations of (**a**) trait anxiety (*r*(92) = −.028, *p* = 1), trait aggression subscales (**b**) physical aggression (*r*(92) = .194, *p* = .304), (**c**) verbal aggression (*r*(92) = −.012, *p* = 1), (**d**) anger (*r*(92) = −.096, *p* = 1), (**e**) mistrust (*r*(92) = .168, *p* = .420), and (**f**) sensation seeking (*r*(92) = .230, *p* = .153) with the amount of retained tokens over all experimental trials. Line indicates linear regression over all data points with 95% confidence interval. Data points are labeled by intervention group: placebo (PP), hydrocortisone (CP), yohimbine (YP), hydrocortisone and yohimbine (CY). *p* values are Bonferroni-Holm corrected for six comparisons

Contrary to our expectations, we found no significant associations between the sum of tokens retained and any personality trait investigated here (see Fig. 5a–f). Nonetheless, the final model of the conducted hierarchical linear regression was a significant predictor of retained tokens (adj. *R*^2^ = 0.691, *F*(24,69) = 9.678, *p* < .001) and included the significant predictors speed when on grid (beta = 350.811, *p* < .001), physical aggression (beta = 19.393, *p* = .022), and the interaction of yohimbine with anger (beta = 18.806, *p* = .032). However, the model held no advantage over the control model, which included gender, age and the average speed on the grid, suggesting that the contributions of the factors physical aggression, and yohimbine in interaction with anger were not substantial (F(21,69) = 0.99, *p* = .477).

### Gender and explorative sex hormone effects

**Fig. 6:**
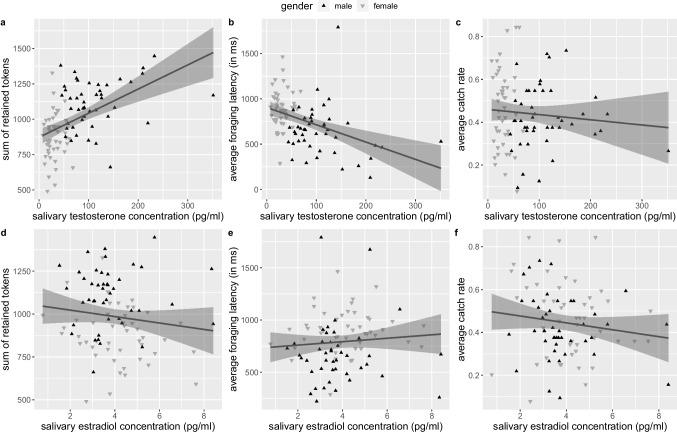
Correlations of the salivary testosterone concentration (pg/ml) with (**a**) the sum of retained tokens over all trials (*r*(90) = .497, *p* < .001), (**b**) the average foraging latency (*r*(90) = −.414, *p* < .001), and (**c**) the average catch rate (*r*(90) = −.087, *p* = .410) as well as the salivary estradiol concentration (pg/ml) with (**d**) retained tokens (*r*(89) = −.134, *p* = .204), (**e**) foraging latency (*r*(89) = .085, *p* = .422), and (**f**) catch rate (*r*(89) = −.141, *p* = .184). Line indicates linear regression over all data points with 95% confidence interval

As expected, men showed overall better task performance. They were caught less often (mean catch rate 38.9% vs. 49.7%, *F*_1,51_=5.980, p=.018, η^2^G=.030), faster to initiate foraging (662 vs. 894 ms, *F*_1,86_=18.310, *p*<.001, η^2^G=.153), and retained more tokens than women (69 vs. 55 per block, *F*_1,86_=39.878, *p*<.001, η^2^G=.135). Testosterone levels also correlated significantly with foraging latency and token-retention (see Fig. 6a–f) and predicted behavior better than respective control models including only age (latency and token-retention: *p*<.001). However, this was not true for the separate-gender-models. No significant effects of estradiol were found.

## Discussion

Stress contributes to the onset and maintenance of several mental disorders in which imbalances in approach-avoidance behaviors play a central role. Investigating how two stress subsystems, e.g., the HPA axis and the SNS, influence approach-avoidance behavior in healthy participants could therefore have important implications for understanding the etiology of stress-related mental disorders. To understand these complex interactions, we investigated the role of cortisol, NA, gender, sex hormones, and personality traits on approach-avoidance behaviors in a foraging-under-threat task, namely the AACT.

### No expected effects of hydrocortisone and yohimbine on approach-avoidance conflict behavior

Despite our successful pharmacological intervention, demonstrated by increased cortisol concentration and alpha amylase activity during task performance, hydrocortisone and yohimbine had very limited effects on approach-avoidance behavior in the AACT. Since the AACT and its manipulations (e.g., threat level and distance) had the expected effects on behavior as demonstrated, for example, by participants’ improved token retention over blocks and ability to distinguish the two predators, it appears that hydrocortisone, yohimbine, and their interaction are not the underlying mechanisms of previously shown stress effects on approach-avoidance behavior (Vogel and Schwabe 2019). While the administration of hydrocortisone and yohimbine cannot imitate something as complex as the stress response with its intricate dynamics of many biological mediators, the associated cognitive appraisal process and subjective impact (Joëls and Baram 2009), we expected stress effects to be at least partially replicated based on previous findings in other cognitive domains.

As the stress network and the receptors for cortisol and NA are present widely across the brain (Joëls and Baram 2009; Ulrich-Lai and Herman 2009), an approximation of stress effects by a pharmacological intervention targeting HPA axis and SNS seemed plausible. However, studies with sample sizes similar to ours utilizing combined hydrocortisone and yohimbine administration in investigation of (other aspects of) human cognition have shown influences of hydrocortisone and yohimbine (e.g., Margittai et al. 2018; Schwabe et al. 2010, 2012; Woodcock et al. 2019; Zerbes et al. 2019), only hydrocortisone (e.g., Kluen et al. 2017a; Kluen et al. 2017c; Metz et al. 2021; Metz et al. 2020), or only yohimbine (e.g., Kausche et al. 2021; Kluen et al. 2017b). The interaction of both is therefore not always at the root of stress-like effects on human cognition in intervention studies. It is further possible that stress-related effects on approach-avoidance behavior in particular are mediated (in part) by different effectors or pathways of the stress response than the ones expected based on the promising memory effects detailed in the introduction, for example, CRH or dopamine (Joëls and Baram 2009). Inverted-U-shape effects of dosage may also play a role (Arnsten 2009) and could be tested in the future by systematically varying the applied dosage. Taken together, it is conceivable that the pharmacological interventions were not effective or specific enough to result in stress-like effects on behavior in approach-avoidance conflicts.

The only significant interaction including one of the pharmacological interventions on our main task measures showed that participants who had not taken yohimbine approached foraging faster when further away from threat than when starting close to the predator. However, faster foraging when away from threat seems counterintuitive as escape from immediate threat (active avoidance; flight) should be faster than approaching the foraging field from the safe place due to response inhibition (passive avoidance; freezing; McNaughton and Corr 2004; Qi et al. 2018). One explanation for the missing but hypothesized threat distance effect in the hydrocortisone/yohimbine group could be that the subjective experience of feeling stressed prior to the AACT is necessary to emphasize the importance of threat at the beginning of each trial. Since our pharmacological intervention did not subjectively affect participants’ mood, the importance of immediate threat may have been underestimated. However, this reasoning would be in contrast to previous studies using comparable pharmacological interventions which likewise reported no mood changes overall while still reporting cognitive effects of the interventions (e.g., Kluen et al. 2017a; Margittai et al. 2016; Putman and Roelofs 2011; Schwabe et al. 2012; please note that we corrected for multiple comparisons, which may have hidden otherwise observable effects). Still, the possibility remains that our results are false-negative, both in the sense that key subjective correlates of harm-avoidance cognition important for anxiety here have not been tapped by our psychometric measures, and in the sense that these measures may have been too noisy. If replicated, effect specificity to yohimbine as noradrenergic drug might be supported by the fact that the systems responsible for avoidance decisions and conflict resolution in approach-avoidance conflicts are innervated by noradrenergic cells of the raphe and locus coeruleus (Gray and McNaughton 2007), potentially opening an avenue for pharmacological manipulation of avoidance behavior.

### No associations between approach-avoidance conflict behavior and personality traits

In contrast to our expectations, we found no associations between the investigated personality traits, i.e., aggression, anxiety, or sensation seeking, and approach-avoidance behavior in the AACT. This is surprising as, e.g., anxiety has been shown to play a central role in approach-avoidance conflicts (Gray and McNaughton 2007). Similarly, there were no differential influences of these personality traits on behavior depending on pharmacological treatment (see also Online Resource 5). This is striking, since underlying differences in observed approach-avoidance behaviors are part of the diagnostic criteria of several anxiety disorders (World Health Organization 1992), strongly indicating that anxious traits should be reflected in task-based approach-avoidance behaviors. Establishing an association between personality traits and approach-avoidance behavior assessed with task-based measures, however, has proven to be difficult (Fricke and Vogel 2020). The AACT has been pharmacologically validated using anxiolytics, e.g., reduced anxiety behavior following lorazepam, valproate, and pregabalin administration (Bach et al. 2018; Korn et al. 2017), suggesting that the task might be sensitive to different levels of anxiety. It has been argued before that the questionnaire employed here (STAI-T) is not specific for anxiety as it also correlates strongly with depressive symptoms (Knowles and Olatunji 2020). However, Bach et al. (2020) also found no correlations of self-reported anxiety (using a different questionnaire) and AACT outcome measures, which was interpreted as the AACT eliciting caution, but not distinguishing stable anxiety levels. Self-reported daringness on the other hand was predictive of approach-avoidance behavior, while sensation seeking in our study was not, which could be interpreted as participants acting daringly, but not recklessly in the AACT. One other reason for our null findings may be that the AACT has been initially constructed to differentiate between groups, which may make it more difficult to draw correlational results by design (Hedge et al. 2018). It may therefore have been more difficult to extract associations of trait anxiety, aggression, and sensation seeking with our behavioral outcome measures.

### Better performance of male participants

Consistent throughout our outcome parameters is the AACT’s sensitivity to gender. Male participants collected more tokens, better avoided getting caught, and started foraging more rapidly. This is in line with Bach et al. (2020) who suggested that preference for economic risk in men (e.g., Lewis et al. 2021), differences in video game experiences and in perception, and experience of threat might explain better performance in men. The larger overestimation of threat in women may be due to general gender differences in threat estimation (Harris and Miller 2000) or specific to the task, could be modifiable by learning, or reflecting life experience, all of which could be investigated in future designs with better, and, more frequent, threat estimation checks. Differences in approach-avoidance behaviors based on gender highlight the need to take participant gender into account as large parts of the variance may be explained by those gender differences. However, approach-avoidance literature to this day often omits gender as potential moderating variable. Nonetheless, our results are not fully representative as women participated based on (non-)use of contraceptives and luteal menstrual cycle phase which limits generalizability. Regarding endogenous testosterone and estradiol, correlative and regression analyses indicate influences of testosterone across gender leading to increased and faster approach behavior. As effects were not present in gender-separated analyses, the effects were likely driven by the general gender performance difference (however, splitting the sample by gender may have resulted in underpowered samples and a large correlation between testosterone levels and gender renders interpretation difficult).

### Limitations and future considerations

The AACT could be an important asset to task-based approach-avoidance research due to the ambiguous conflict it creates compared with other approach-avoidance tasks with clearly instructed correct responses (e.g., Chen and Bargh 1999; De Houwer et al. 2001). The caveat of this ambiguity is the impossibility to determine whether approach or avoidance is the driving force of behavior. A future distinction of approach and avoidance may be helpful since the underlying biological systems of approach, avoidance, and conflict sensitivity are theorized to be distinct, but interacting (Gray and McNaughton 2007). Future research on behavior in approach-avoidance conflicts should therefore consider how the systems can be measured (and experimentally manipulated) separately, while still keeping the decision space within the task broader than classical approach-avoidance tasks.

A major advantage of the AACT is that threat level and initial threat distance can be easily manipulated. However, we were unable to confirm our assumption that a short (vs. long) initial threat distance would lead to faster initial responding. Perhaps perceived threat distance was amplified by the temporal distance to threat as there was at least a 6-s window before predator wake-up and therefore enough time to escape. Moreover, the experience of threat level can also be based on the speed with which a predator becomes active (Fung et al. 2019). The differential effects of threat distance under stress may thus be partially attributed to changes in temporal processing. While stress is usually associated with the feeling of “time slowing down” (van Hedger et al. 2017), anxiety towards unpredictable events such as threat wake-up was also reported to accelerate time perception (Sarigiannidis et al. 2020), which could lead to a greater feeling of imminent threat under stress. It should thus be considered to implement shorter latencies of predator wake-up in the future to make the unpredictable, imminent nature of the threat more present. On a final note, since another risk-taking task (BART) had been performed twice before the AACT, participants may have habituated to risk-taking in general and therefore been less sensitive to the AACT manipulations. As the tasks are very different in nature, this might not be likely, but nonetheless task-order-randomization in future studies would be advisable.

In conclusion, our hypotheses that the combined effects of cortisol and NA would resemble previously reported stress effects on approach-avoidance behavior did not hold true. We believe the most natural explanation would be that cortisol and NA are either not the (sole) relevant mediators for changes in approach-avoidance behaviors under stress, requiring further aspects of the stress response to be active, or that dosage of the interventions needed to be more fine-tuned. Gender, however, affects almost all outcome parameters investigated here. This stresses the importance of properly controlling for or specifically investigating gender in approach-avoidance research in the future.

## Supplementary information

ESM 1 Online Resource (DOCX 526 kb)

## Data Availability

All analyses were conducted in R (Version 4.2.2) and can be found at osf.io/d69pr. The data is made available at osf.io/d69pr within the analysis structure.
