# Supplementary material for: The effects of hydrocortisone and yohimbine on human behavior in approach-avoidance conflicts
Source: Psychopharmacology (Berl). 2023 Jun 14;240(8):1705–17. doi: 10.1007/s00213-023-06396-6 (PMC10265560; doi:10.1007/s00213-023-06396-6)
Supplement: Supplementary file 1 — Online Resource (DOCX 526 kb) [file 213_2023_6396_MOESM1_ESM.docx]

**Online Resource 1: Detailed inclusion and exclusion criteria**

Participants had to be between the ages of 18 and 35 years with normal or corrected-to-normal vision, normal body weight (body mass index: 18.5 to 26.5 kg/m²), a regular sleep-wake cycle, German as mother tongue or equivalent proficiency and naïve to both experimental tasks. Participant’s health status was assessed in a standardized telephone screening prior to participation. Individuals were excluded if they had any physical, psychiatric, neurological, endocrine, cardiovascular or internal conditions, osteoporosis, asthma or glaucoma. Individuals were further excluded if they had been vaccinated within the last month, had used medication within the last month affecting stress reactivity or reported other events that potentially affected their stress response. Drinking more than 15 glasses of wine (or an equivalent alcohol consumption) per week as well as prior or past illicit drug use led to exclusion. An exception was made for cannabis use if at least 2 months in the past and with a frequency below once per month as well as the singular consumption of other illicit drugs more than 6 months ago without withdrawal symptoms. To keep hypothalamus-pituitary-adrenal (HPA) axis responsiveness comparable, smokers were excluded if they smoked more than five cigarettes a week (Rohleder & Kirschbaum, 2006). Similarly, pregnant or breastfeeding women as well as women on hormonal contraceptives within the last 3 months were excluded. Women additionally needed to have a regular menstrual cycle and were scheduled for the laboratory visit during their luteal phase in order to avoid effects of the menstrual cycle (Kirschbaum, Kudielka, Gaab, Schommer, & Hellhammer, 1999).

Participants were recruited via advertisement at our university as well as through an online recruitment platform and included both students and non-students. Two male participants were excluded from all analyses due to fulfilling exclusion criteria (determined in post-experiment conversation and initially not disclosed) and displaying non-compliance during the study, respectively, leading to a total sample size of n = 94 out of 96 initially recruited.

**Online Resource 2: Vital sign measures and saliva sampling**

Vital signs were measured with an automated wrist monitor (RS2, OMRON, the Netherlands). Each assessment was performed twice and mean values were used for analyses. Saliva was collected via Salivettes® (Sarstedt, Germany) to assess cortisol concentration and alpha amylase activity. In total, vital signs and saliva via Salivettes were collected five times throughout the experiment. In addition, native saliva was collected via microtubes with a straw for the assessment of testosterone and estrogen concentrations (directly after the first and fourth collection of saliva via Salivette). Collection time of native saliva varied based on the participants’ ability to produce native saliva.

The saliva samples were frozen and stored at -18 °C (-0.4 °F) immediately after testing and later moved to a -80 °C (-112 °F) unit for long-term storage until shipping to Dresden Lab Service GmbH for analysis. After thawing, Salivettes were centrifuged at 3,000 rpm for 5 min, which resulted in a clear supernatant of low viscosity. Salivary cortisol concentrations were measured using commercially available chemiluminescence immunoassay with high sensitivity (IBL International, Hamburg, Germany). The intra- and interassay coefficients were below 4.3% and 5%, respectively. Activity of salivary alpha-amylase was measured by an enzyme kinetic method as described by Rohleder et al. (2006). The intra- and interassay coefficients were below 4.1% and 4.4%, respectively. Participants for which at least one sample did not contain sufficient saliva were excluded from analyses involving salivary cortisol (n=6; placebo: n=2, hydrocortisone: n=2, yohimbine: n=1, hydrocortisone and yohimbine: n=1) or salivary alpha-amylase (n=8; placebo: n=3, hydrocortisone: n=2, yohimbine: n=2, hydrocortisone and yohimbine: n=1), respectively.

The native saliva samples were similarly frozen and stored at -18 °C (-0.4 °F) immediately after testing and later moved to a -80 °C (-112 °F) unit for long-term storage until shipping to Dresden Lab Service GmbH for analysis. After thawing, samples were centrifuged at 12,000 rpm for 5 min and 200 µL supernatant injected into a liquid chromatography–tandem mass spectrometry system to determine testosterone and estradiol concentrations as described in Gao et al. (2015). Intra-and inter-assay coefficients of variance were between 4.3% and 10.8%.

**Online Resource 3: Detailed description of the approach-avoidance task and additional outcome parameters investigated**

*Approach-Avoidance conflict task (AACT)*

To assess approach and avoidance behavior, we employed an adapted version of the AACT (programmed in Python 3.2.5 using Pygame 1.9.2; see Vogel and Schwabe 2019), originally developed by Bach et al. (2014; see Figure 1). The task was inspired by anxiety research in rodents to investigate human behavior in approach-avoidance conflicts and induced behavioral inhibition and passive avoidance as shown before in analogue tasks in non-human animals (Bach et al. 2014; for a more general discussion on the use of non-human behavioral paradigms in psychiatric research, see for instance: Geyer and Markou 1995; Shemesh and Chen 2023) . Moreover, the task was pharmacologically validated using anxiolytics such that intake of lorazepam, valproate or pregabalin reduced anxious behavior in approach-avoidance conflicts (Bach et al. 2018; Korn et al. 2017).

The AACT is trial-based and played on a 24 x 16 grid presented on a standard computer monitor. The grid contained 1) a player-controlled green triangle, 2) 10 diamond-shaped yellow tokens of which one randomly changed location every two seconds (and a new token appeared in a random location if participants collected one), and 3) a predator (initially a grey circle) that could wake up during a trial to chase the player and was initially located in a randomly chosen corner opposite to 4) a black square, representing a safe space in which the player could not be caught by the predator. The green triangle was controlled with the arrow keys of a keyboard to a maximum speed of 8 blocks/s, whereas the predator was at minimum 2.5 times faster than the player at a constant 20 blocks/s. Therefore, escaping the awoken predator was only possible if participants were in the proximity of the safe place. Participants were instructed to collect as many tokens as possible without being caught by the predator as random trials would be selected to determine additional monetary compensation.

Trials could differ on several accounts. Participants could start either in the safe place (50% of trials; passive avoidance to stay away from threat) or in the same location as the predator (active avoidance to escape the threat first and passive avoidance later). Threat level could be high (50% of trials; 60% chance for the predator to wake up) or low (20% chance), and was represented by the initial grid border color (orange or blue; color-threat level association was randomized over participants), but not explicitly instructed. The predator assumed the color of the border in case of wake-up, and the border turned red to clearly indicate the active threat. Trials without predator wake-up could last 6, 7.5, 9, 10.5, 12, 13.5, or 15 s. For trials with active predator, the predator woke up after the same durations and had to be avoided for 3.5 seconds. These trials ended either after 3.5 seconds or when the player was caught beforehand.

In total, participants played 160 trials, evenly divided into four blocks and separated by self-paced breaks, taking 42 minutes on average. The 50% split between the two threat levels and starting positions was maintained within each block as well as the distribution of trial durations. Due to a programming error, however, threat level and threat distance were not balanced in regard to each other. This led to task blocks in which there were missing combinations of threat level x threat distance x wake-up of threat for individual participants. After the AACT, participants were asked to estimate the wake-up probabilities for both predators.

*Supplemental outcome parameters*

Recently, Bach et al. (Bach et al. 2020) assessed test-retest reliability of AACT outcome variables over 11-32 months in 567 participants (aged 14-24 years). Several variables reached a sufficient test-retest coefficient of above 0.6: average number of tokens retained after predator wake-up (or end of trial if predator did not wake up; 0.689), average token collection (0.686), decrease in token collection over time in trial (0.771), average speed when on grid (0.681), and decrease in speed when on grid (0.608). Of note, no interactions with threat level displayed sufficient test-retest reliability (all coefficients < 0.173). Given these findings, we included the remaining four of these five variables with high test-retest reliability (tokens retained was covered in our primary analyses) in additional analyses to enhance comparability with the work of Bach and colleagues. Finally, we also included mixed-design ANOVAs for six outcome variables over time-in-trial (e.g., time spent in safe space; speed; token collection rate; see Supplement-Specific Figure SS2 and Table SS1) to enhance comparability with previous findings (e.g., Bach et al. 2014; Bach et al. 2018; Korn et al. 2017).

**Online Resource 4: Influence of time of recruitment on participant characteristics**

*Analysis*

Given that recruitment was spread over a longer time period, which contained the onset of the Covid-19 pandemic, we explored whether our recruitment might be biased over time such that only certain individuals partook in our study as the pandemic progressed. To this end, we explored trait anxiety and sensation seeking scores over time of recruitment in linear regressions.

*Results*


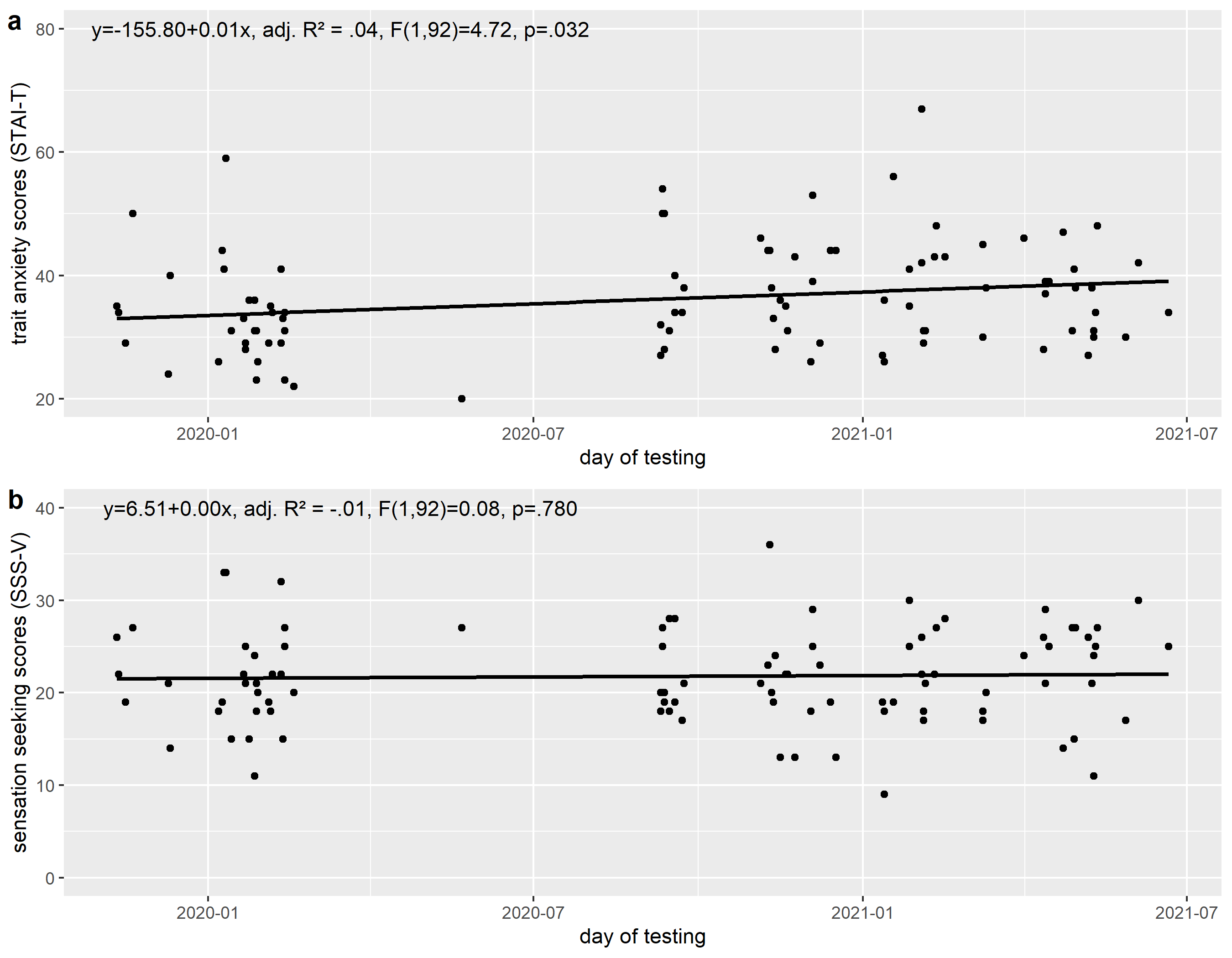
Regarding changes in sample composition over time (experimental period: 11.11.2019 to 21.06.2021) as the pandemic developed and progressed, there was a slight increase in trait anxiety scores (adj. R² = .04, F(1,92) = 4.72, p = .032), while sensation seeking scores did not change (adj. R² = -.01, F(1,92) = 0.08, p = .780), suggesting that the covid-19 pandemic had no major effects on the type of participants who volunteered for our experiment (see Supplement-specific Figure SS1).

**Fig SS1**

Trait anxiety scores (a) and sensation seeking scores (b) of participants over day of testing to see if participant population was influenced by covid-19 pandemic onset

**Online Resource 5: Analyses and results of additional test-retest reliable parameters and time-over-trial parameters**

Due to the rich possibilities in parameterization of the AACT, it is possible that relevant outcome parameters have been missed in our main analyses. Therefore, we investigate both additional test-retest reliable parameters and time-over-trial parameters in this section. For future research, it will be vital to understand which approach-avoidance task features index which cognitive component, are comparable with other tasks and potentially link with personality or psychiatric disorders. This would aid in the reconciliation of ambiguous findings in the current approach-avoidance literature and thus allow better understanding of underlying cognitive processes.

*Test-retest reliable Parameters:*

*Analysis*

For the parameters with high test-retest reliability identified above (decrease in token collection, decrease in speed, average speed, average token collection; Bach et al. 2020) – see Online Resource 3, we conducted mixed-design ANOVAs with the within-subject factor initial threat distance (long vs. short) and the between-subject factors hydrocortisone, yohimbine and gender. Bonferroni-Holm correction was applied for four outcome variables. Initial threat distance was included due to a recent study reporting the dependence of stress effects in task-based approach-avoidance to depend on it (Vogel and Schwabe 2019).

We additionally carried out linear hierarchical regressions as described for the sum of tokens retained in the main text for the high test-retest reliable parameters aside from removing speed on grid as predictor, since two of the variables were directly related to speed during the AACT. In blockwise fashion, the following mean-centered variables were introduced: 1) The control variables gender, age, followed by 2) the interventions (hydrocortisone, yohimbine) as well as their interaction, 3) the personality trait measures (trait anxiety by STAI-T total score (Laux et al. 1981), sensation seeking by SSSV total score (Beauducel et al. 2003) and aspects of trait aggression by four subscales of the DAF, namely physical aggression, verbal aggression, anger and mistrust (Werner and von Collani 2014)), and finally 4) the interactions of personality trait measures with the interventions.

*Results -* *Test-retest reliable parameters are not influenced by hydrocortisone or yohimbine*

For the test-retest reliable parameters (aside retained tokens, which has been covered in the main text), ANOVAs focusing on the initial distance of threat due to the importance of threat distance for approach-avoidance behavior in recent studies (Fung et al. 2019; Vogel and Schwabe 2019) were conducted. We found effects of gender as male participants collected more tokens on average (average token collection per 500ms: 0.37 vs. 0.303; F(1,86) = 27.663, p < 0.001, η^2^G = 0.238) and kept a higher average speed compared to female participants (average speed per 500ms: 2.99 vs. 2.50; F(1,86) = 40.702, p < 0.001, η^2^G = 0.318). Initial threat distance also mattered as starting in the immediate vicinity of the predator led to increases in both, average token collection (starting location: 0.348 vs. 0.323.; F(1,86) = 122.806, p < 0.001, η^2^G = 0.04) and slope of the token collection (starting location: 0.0000167 vs. 0.0000121; F(1,86) = 70.555, p < 0.001, η^2^G = 0.066). No significant effects were found for the slope of speed. However, yohimbine and hydrocortisone did not show any significant main effects or interactions for any of the four parameters.

Regarding the hierarchical regression models for the test-retest reliable parameters, none of the regression models including personality traits, intervention or their interactions performed better than their respective control models, indicating no advantage of adding those effects to the model.

*Time-Over-Trial Parameters:*

As seen in previous studies and to enhance comparability (e.g., Bach et al. 2014; Bach et al. 2018; Korn et al. 2017), we also included mixed-design ANOVAs for six outcome variables over time-in-trial. To analyze intervention effects on behavior over time-in-trial we used ANOVAs as described for our summary variables in the main text, and included the factor time-in-trial but omitted the factor block. Bonferroni-Holm correction was applied for six outcome variables. Supplement-specific Figure SS2 displays the trajectories of the averaged trials in each of the groups divided by starting close or far away from threat. The supplement-specific Table SS1 (placed at the end of the document due to its length) displays the results of the Over-Trial ANOVAs. Please note that these analyses revealed no or minimal effects of yohimbine and hydrocortisone on behavior over time-in-trial also.


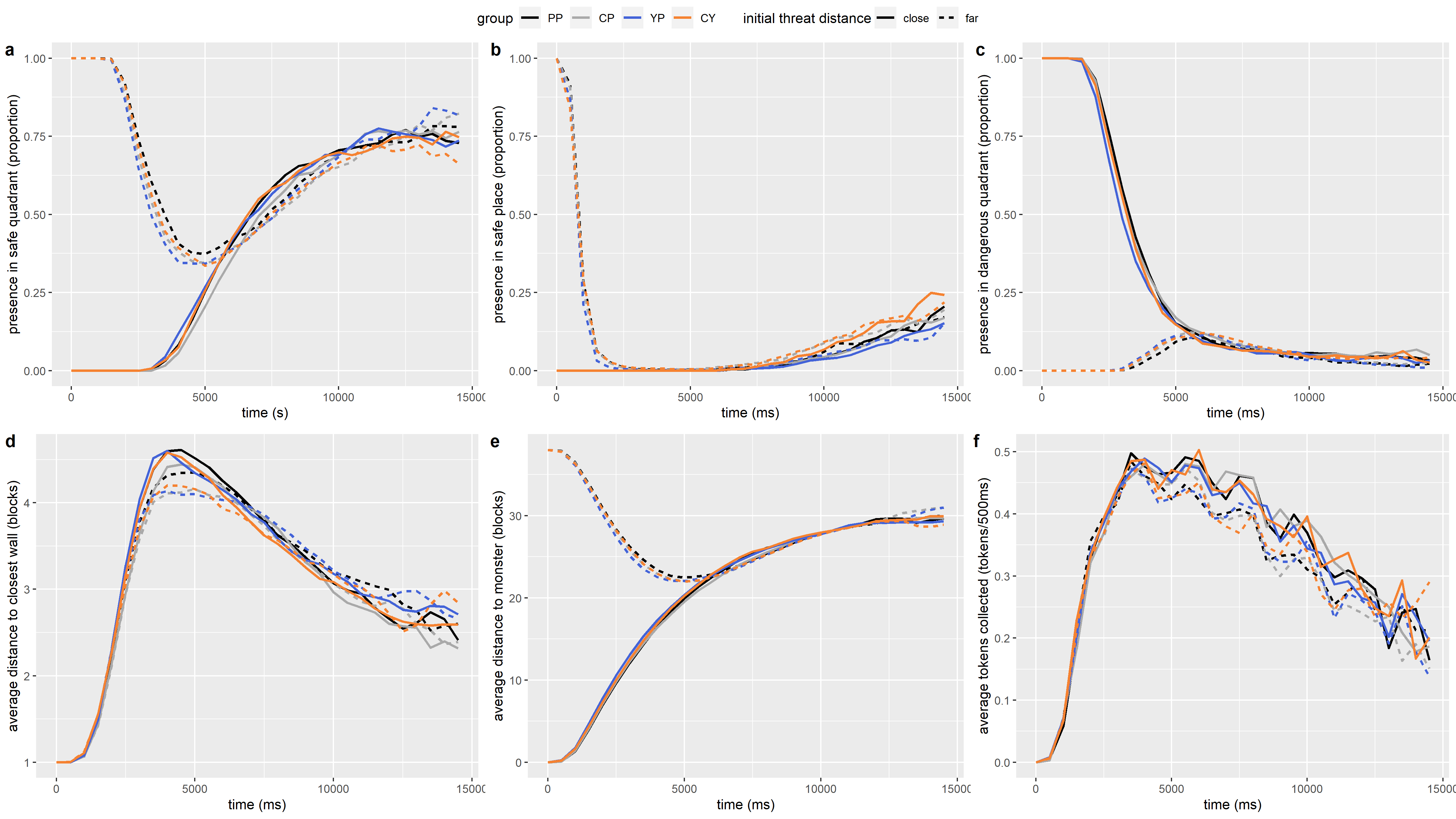


**Fig SS2**

Averaged measurements over time in trial (each 500ms) for (a) presence in safe quadrant, (b) presence in safe place, (c) presence in dangerous quadrant, (d) average distance to closest wall, (e) average distance to predator and (f) average tokens collected, split according to initial threat distance. Groups: placebo (PP), hydrocortisone (CP), yohimbine (YP), hydrocortisone and yohimbine (CY)

**Online Resource 6: Analyses and results regarding the influences of threat overestimation on summary outcome measures**

In the results section Participants overestimate low threat condition in the approach-avoidance conflict task, we noted that women overestimated threat more than men (mean overestimation of wake-up probability 12.0% vs. 7.7%, F1,86=5.454, p=.022, η2G=.019, see Online Resource Figure S1). As advised by a reviewer, it is interesting to exploratively investigate in how far threat overestimation predicts our summary outcome measures and if this is based on either gender or testosterone effects. To this end, we first correlated gender, testosterone, threat overestimation and our summary outcome variables (sum of collected tokens, average foraging latency, catch rate). The correlations can be found in Table SS2. Average threat overestimation was moderately correlated to two outcome variables, such that individuals who overestimated threat more retained less tokens and were more inhibited (i.e. had longer foraging latencies).

We additionally carried out linear hierarchical regressions for all three summary variables, once with and once without gender and testosterone added as control variables. In blockwise fashion, the following mean-centered variables were introduced: 1) The control variables age and speed during the task and optionally gender and basal testosterone level, followed by 2) the individual overestimation of threat (averaged over high and low threat conditions). These regressions did not show any advantage of adding the average overestimation to the model independent of whether gender and testosterone were included or not. Further, speed during the task was the only significant predictor in all models. For readers that are interested in further analyses, we recommend exploring the data set as well as the accompanying analysis files available at https://osf.io/d69pr/.

Table SS2. Correlations of threat overestimation, gender, testosterone level and outcome measures

|  | 1 | 2 | 3 | 4 | 5 | 6 | 7 | 8 |
| --- | --- | --- | --- | --- | --- | --- | --- | --- |
| 1. gender | - |  |  |  |  |  |  |  |
| 2. testosterone | .68** | - |  |  |  |  |  |  |
| 3. overestimation (average) | -.23* | -.23* | - |  |  |  |  |  |
| 4. overestimation (high-threat) | -.22* | -.09 | .64** | - |  |  |  |  |
| 5. overestimation (low-threat) | -.03 | -.18 | .51** | -.33** | - |  |  |  |
| 6. sum of collected tokens | .58** | .50** | -.31** | -.09 | -.28** | - |  |  |
| 7. average foraging latency | -.47** | -.41** | .33** | .09 | .30** | -.73** | - |  |
| 8. catch rate | -.13 | -.09 | -.03 | -.01 | -.03 | -.10 | .01 | - |

Note. Gender coding: woman = 0 and man = 1. * p <.05. ** p <.01.

**Supplemental Figures**


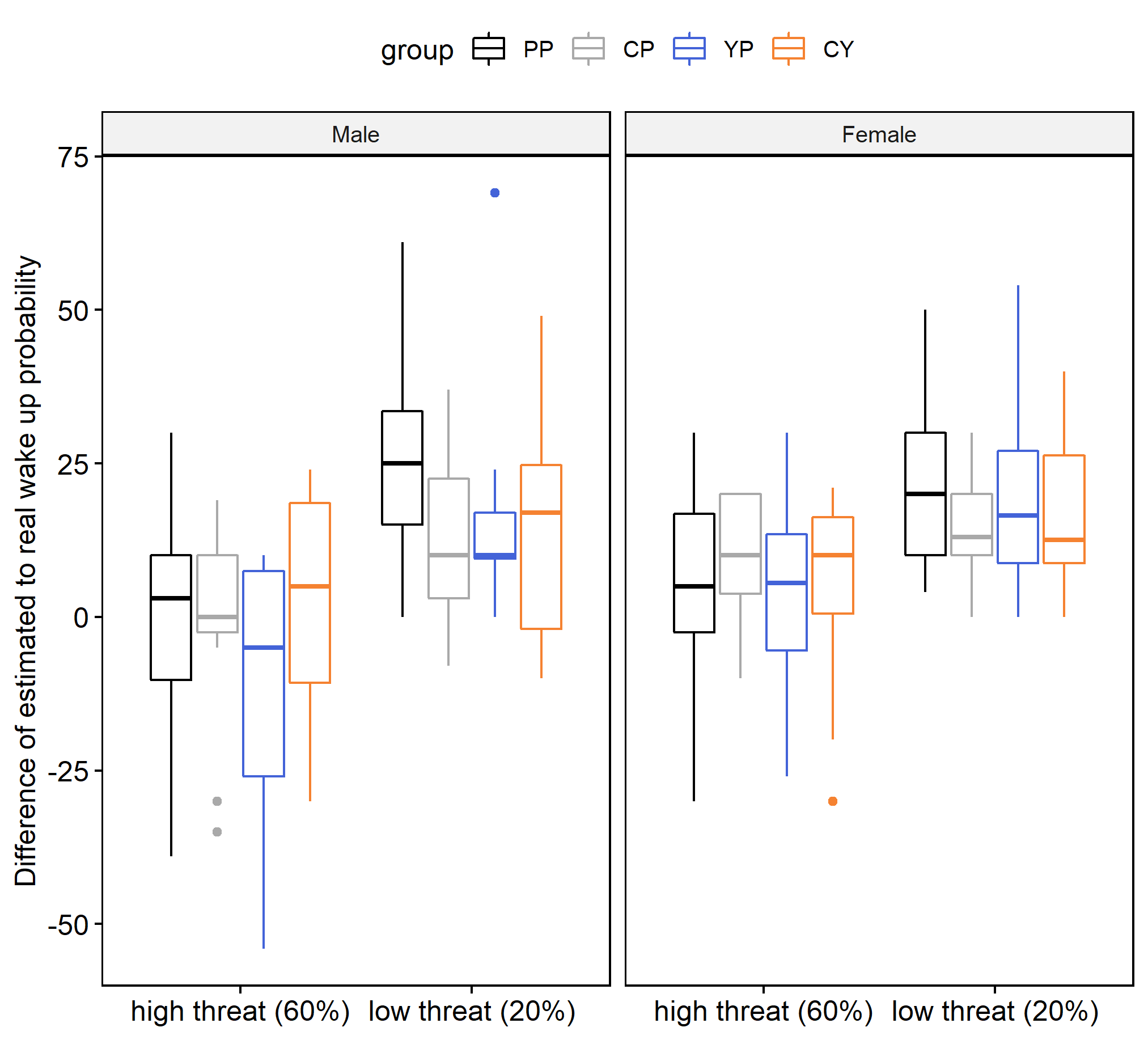


**Fig S1**

Differences in over-/underestimation of wake-up rates based on pharmacological intervention and gender. Groups: placebo (PP), hydrocortisone (CP), yohimbine (YP), hydrocortisone and yohimbine (CY)

**Supplementary Tables**

Table S1. Sample Characteristics

|  | PP (N=24) | CP (N=23) | YP (N=23) | CY (N=24) | F-value | p value |
| --- | --- | --- | --- | --- | --- | --- |
| **age** |  |  |  |  | 0.764 | .517^1^ |
| Mean (SD) | 25.42 (5.12) | 24.78 (4.24) | 23.57 (3.62) | 24.25 (4.30) |  |  |
| Lower CI (95%) | 23.25 | 22.95 | 22.00 | 22.44 |  |  |
| Upper CI (95%) | 27.58 | 26.62 | 25.13 | 26.06 |  |  |
| **BMI** |  |  |  |  | 0.945 | .423^1^ |
| Mean (SD) | 23.05 (2.12) | 22.59 (2.48) | 22.38 (1.81) | 22.07 (1.82) |  |  |
| Lower CI (95%) | 22.16 | 21.52 | 21.59 | 21.31 |  |  |
| Upper CI (95%) | 23.95 | 23.66 | 23.16 | 22.84 |  |  |
| **trait anxiety** |  |  |  |  | 0.651 | .584^1^ |
| Mean (SD) | 34.54 (7.19) | 38.04 (9.10) | 36.61 (10.22) | 35.88 (8.40) |  |  |
| Lower CI (95%) | 31.51 | 34.11 | 32.19 | 32.33 |  |  |
| Upper CI (95%) | 37.58 | 41.98 | 41.03 | 39.42 |  |  |
| **physical aggression** |  |  |  |  | 0.468 | .705^1^ |
| Mean (SD) | 13.17 (3.60) | 14.22 (3.91) | 13.09 (3.13) | 13.29 (4.04) |  |  |
| Lower CI (95%) | 11.65 | 12.53 | 11.73 | 11.59 |  |  |
| Upper CI (95%) | 14.68 | 15.91 | 14.44 | 15.00 |  |  |
| **verbal aggression** |  |  |  |  | 0.969 | .411^1^ |
| Mean (SD) | 10.04 (2.46) | 10.96 (2.6) | 11.35 (3.02) | 10.79 (2.7) |  |  |
| Lower CI (95%) | 9.00 | 9.83 | 10.04 | 9.65 |  |  |
| Upper CI (95%) | 11.08 | 12.08 | 12.66 | 11.93 |  |  |
| **anger** |  |  |  |  | 0.104 | .957^1^ |
| Mean (SD) | 12.29 (3.61) | 12.83 (3.83) | 12.43 (3.42) | 12.71 (3.85) |  |  |
| Lower CI (95%) | 10.77 | 11.17 | 10.95 | 11.08 |  |  |
| Upper CI (95%) | 13.81 | 14.48 | 13.91 | 14.33 |  |  |
| **mistrust** |  |  |  |  | 0.617 | .606^1^ |
| Mean (SD) | 13.08 (4.09) | 14.30 (3.69) | 14.26 (3.51) | 13.54 (3.27) |  |  |
| Lower CI (95%) | 11.36 | 12.71 | 12.74 | 12.16 |  |  |
| Upper CI (95%) | 14.81 | 15.90 | 15.78 | 14.92 |  |  |
| **sensation seeking** |  |  |  |  | 1.496 | .221^1^ |
| Mean (SD) | 20.00 (6.34) | 22.35 (5.22) | 21.70 (4.91) | 23.04 (4.11) |  |  |
| Lower CI (95%) | 17.32 | 20.09 | 19.57 | 21.31 |  |  |
| Upper CI (95%) | 22.68 | 24.60 | 23.82 | 24.78 |  |  |

Note. Degrees of freedom are 3 and 90 for all tests. Groups: placebo (PP), hydrocortisone (CP), yohimbine (YP), hydrocortisone and yohimbine (CY). ^1^Linear Model ANOVA.

*Table S2. Characteristics of MDBF and VAS*

|  | PP (N=24) | CP (N=23) | YP (N=23) | CY (N=24) |
| --- | --- | --- | --- | --- |
| **MDBF awake-tired 1** |  |  |  |  |
| Mean (CI) | 16.333 (15.644, 17.023) | 15.609 (14.576, 16.641) | 15.739 (14.744, 16.734) | 15.833 (14.736, 16.931) |
| **MDBF awake-tired 2** |  |  |  |  |
| Mean (CI) | 15.167 (14.091, 16.243) | 15.348 (14.199, 16.496) | 14.739 (13.586, 15.892) | 14.458 (13.135, 15.782) |
| **MDBF awake-tired 3** |  |  |  |  |
| Mean (CI) | 13.875 (12.718, 15.032) | 13.609 (12.294, 14.924) | 13.348 (11.824, 14.872) | 13.792 (12.232, 15.352) |
| **MDBF calm-restless 1** |  |  |  |  |
| Mean (CI) | 16.542 (15.697, 17.386) | 15.870 (14.513, 17.226) | 16.000 (14.709, 17.291) | 15.917 (14.906, 16.928) |
| **MDBF calm-restless 2** |  |  |  |  |
| Mean (CI) | 16.292 (14.886, 17.698) | 16.348 (14.910, 17.786) | 14.261 (12.207, 16.315) | 14.875 (13.222, 16.528) |
| **MDBF calm-restless 3** |  |  |  |  |
| Mean (CI) | 15.792 (14.242, 17.342) | 14.913 (13.473, 16.353) | 13.261 (11.393, 15.129) | 13.208 (11.580, 14.837) |
| **MDBF good-bad 1** |  |  |  |  |
| Mean (CI) | 17.375 (16.479, 18.271) | 16.652 (15.689, 17.616) | 17.043 (16.108, 17.979) | 17.000 (16.085, 17.915) |
| **MDBF good-bad 2** |  |  |  |  |
| Mean (CI) | 17.250 (16.422, 18.078) | 16.783 (15.708, 17.857) | 16.217 (14.826, 17.609) | 16.083 (14.569, 17.598) |
| **MDBF good-bad 3** |  |  |  |  |
| Mean (CI) | 16.917 (16.011, 17.822) | 16.652 (15.411, 17.893) | 16.304 (14.891, 17.717) | 16.125 (14.645, 17.605) |
| **VAS anxious 1** |  |  |  |  |
| Mean (CI) | 1.375 (1.102, 1.648) | 1.826 (1.219, 2.433) | 1.870 (1.312, 2.427) | 1.458 (1.210, 1.707) |
| **VAS anxious 2** |  |  |  |  |
| Mean (CI) | 1.333 (1.095, 1.572) | 1.304 (1.063, 1.546) | 1.652 (1.046, 2.258) | 1.417 (1.141, 1.693) |
| **VAS anxious 3** |  |  |  |  |
| Mean (CI) | 1.333 (1.012, 1.655) | 1.304 (0.974, 1.635) | 1.826 (1.005, 2.647) | 1.417 (1.141, 1.693) |
| **VAS upset 1** |  |  |  |  |
| Mean (CI) | 2.708 (2.145, 3.272) | 2.783 (2.144, 3.421) | 3.000 (2.298, 3.702) | 2.542 (2.060, 3.024) |
| **VAS upset 2** |  |  |  |  |
| Mean (CI) | 2.125 (1.592, 2.658) | 1.783 (1.371, 2.194) | 2.696 (1.690, 3.701) | 2.208 (1.743, 2.674) |
| **VAS upset 3** |  |  |  |  |
| Mean (CI) | 1.875 (1.388, 2.362) | 2.435 (1.772, 3.097) | 2.739 (1.965, 3.513) | 2.708 (1.916, 3.501) |
| **VAS stressed 1** |  |  |  |  |
| Mean (CI) | 1.792 (1.463, 2.121) | 2.565 (1.763, 3.367) | 2.043 (1.398, 2.689) | 2.167 (1.500, 2.833) |
| **VAS stressed 2** |  |  |  |  |
| Mean (CI) | 1.792 (1.279, 2.305) | 1.826 (1.192, 2.460) | 2.304 (1.465, 3.144) | 2.000 (1.472, 2.528) |
| **VAS stressed 3** |  |  |  |  |
| Mean (CI) | 2.000 (1.472, 2.528) | 2.217 (1.730, 2.704) | 2.696 (1.846, 3.545) | 2.542 (1.618, 3.465) |

Note. Average and 95% confidence interval of the Mehrdimensionaler Befindlichkeitsfragebogen (MDBF (Steyer et al. 1994)) and three visual analogue scales. Bonferroni-Holm corrected ANOVAs for 6 variables with the between-variables hydrocortisone and yohimbine and the within-variable time point (1: at the begin of the experiment, 2: 45 minutes after intake of medication, or 3: at end of experiment) did not show changes in mood based on pharmacological intervention. Across groups, MDBF awake-tired (F(1.81, 162.82) = 28.428, p < .001, η^2^G = .100), MDBF calm-restless (F(2, 180) = 13.319, p < .001, η^2^G = .045) and VAS upset (F(2, 180) = 5.684, p = .016, η^2^G = .023) significantly varied over time points. Groups: placebo (PP), hydrocortisone (CP), yohimbine (YP), hydrocortisone and yohimbine (CY).

*Table SS1. Results of Over-Trial Outcome Variable ANOVAs*

| **Effect** | **Outcome Variable** | **F-value with DF(n,d)** | **p** | **p<.05** | **p.adj** | **p.adj**  **<.05** | **ges** |
| --- | --- | --- | --- | --- | --- | --- | --- |
| hydrocortisone | tokens collected | F(1,84)=0 | 0.994 |  | 1.000 |  | <0.001 |
| hydrocortisone:threatlevel | tokens collected | F(1,84)=0.082 | 0.775 |  | 1.000 |  | <0.001 |
| hydrocortisone:threatdistance | tokens collected | F(1,84)=0.711 | 0.401 |  | 1.000 |  | <0.001 |
| hydrocortisone:threatdistance:threatlevel | tokens collected | F(1,84)=1.484 | 0.227 |  | 1.000 |  | <0.001 |
| hydrocortisone:gender | tokens collected | F(1,84)=0 | 0.992 |  | 1.000 |  | <0.001 |
| hydrocortisone:gender:threatlevel | tokens collected | F(1,84)=0.19 | 0.664 |  | 1.000 |  | <0.001 |
| hydrocortisone:gender:threatdistance | tokens collected | F(1,84)=0.187 | 0.667 |  | 1.000 |  | <0.001 |
| hydrocortisone:gender:threatdistance:threatlevel | tokens collected | F(1,84)=0.484 | 0.488 |  | 1.000 |  | <0.001 |
| hydrocortisone:gender:time | tokens collected | F(9.03,758.43)=0.385 | 0.943 |  | 1.000 |  | 0.001 |
| hydrocortisone:gender:time:threatlevel | tokens collected | F(10.76,903.57)=1.413 | 0.163 |  | 0.978 |  | 0.003 |
| hydrocortisone:gender:time:threatdistance | tokens collected | F(13.32,1118.94)=0.663 | 0.804 |  | 1.000 |  | 0.001 |
| hydrocortisone:gender:time:threatdistance:threatlevel | tokens collected | F(13.18,1107.26)=0.583 | 0.871 |  | 1.000 |  | 0.001 |
| hydrocortisone:time | tokens collected | F(9.03,758.43)=0.72 | 0.692 |  | 1.000 |  | 0.002 |
| hydrocortisone:time:threatlevel | tokens collected | F(10.76,903.57)=1.228 | 0.264 |  | 0.792 |  | 0.003 |
| hydrocortisone:time:threatdistance | tokens collected | F(13.32,1118.94)=1.483 | 0.114 |  | 0.684 |  | 0.003 |
| hydrocortisone:time:threatdistance:threatlevel | tokens collected | F(13.18,1107.26)=0.788 | 0.676 |  | 1.000 |  | 0.001 |
| hydrocortisone:yohimbine | tokens collected | F(1,84)=0.069 | 0.793 |  | 1.000 |  | <0.001 |
| hydrocortisone:yohimbine:threatlevel | tokens collected | F(1,84)=0.095 | 0.759 |  | 1.000 |  | <0.001 |
| hydrocortisone:yohimbine:threatdistance | tokens collected | F(1,84)=2.496 | 0.118 |  | 0.708 |  | <0.001 |
| hydrocortisone:yohimbine:threatdistance:threatlevel | tokens collected | F(1,84)=0.085 | 0.771 |  | 1.000 |  | <0.001 |
| hydrocortisone:yohimbine:gender | tokens collected | F(1,84)=0.609 | 0.437 |  | 1.000 |  | 0.002 |
| hydrocortisone:yohimbine:gender:threatlevel | tokens collected | F(1,84)=3.574 | 0.062 |  | 0.372 |  | 0.001 |
| hydrocortisone:yohimbine:gender:threatdistance | tokens collected | F(1,84)=2.188 | 0.143 |  | 0.715 |  | <0.001 |
| hydrocortisone:yohimbine:gender:threatdistance:threatlevel | tokens collected | F(1,84)=2.164 | 0.145 |  | 0.435 |  | <0.001 |
| hydrocortisone:yohimbine:gender:time | tokens collected | F(9.03,758.43)=1.276 | 0.246 |  | 1.000 |  | 0.004 |
| hydrocortisone:yohimbine:gender:time:threatlevel | tokens collected | F(10.76,903.57)=0.494 | 0.905 |  | 1.000 |  | 0.001 |
| hydrocortisone:yohimbine:gender:time:threatdistance | tokens collected | F(13.32,1118.94)=1.783 | 0.040 | * | 0.200 |  | 0.003 |
| hydrocortisone:yohimbine:gender:time:threatdistance:threatlevel | tokens collected | F(13.18,1107.26)=1.167 | 0.298 |  | 0.894 |  | 0.002 |
| hydrocortisone:yohimbine:time | tokens collected | F(9.03,758.43)=1.027 | 0.417 |  | 1.000 |  | 0.003 |
| hydrocortisone:yohimbine:time:threatlevel | tokens collected | F(10.76,903.57)=1.38 | 0.179 |  | 1.000 |  | 0.003 |
| hydrocortisone:yohimbine:time:threatdistance | tokens collected | F(13.32,1118.94)=1.485 | 0.114 |  | 0.342 |  | 0.003 |
| hydrocortisone:yohimbine:time:threatdistance:threatlevel | tokens collected | F(13.18,1107.26)=0.955 | 0.495 |  | 1.000 |  | 0.002 |
| threatlevel | tokens collected | F(1,84)=15.34 | <0.001 | * | 0.001 | * | 0.003 |
| threatdistance | tokens collected | F(1,84)=83.246 | <0.001 | * | <0.001 | * | 0.007 |
| threatdistance:threatlevel | tokens collected | F(1,84)=1.556 | 0.216 |  | 0.432 |  | <0.001 |
| gender | tokens collected | F(1,84)=14.406 | <0.001 | * | 0.002 | * | 0.036 |
| gender:threatlevel | tokens collected | F(1,84)=0.639 | 0.426 |  | 1.000 |  | <0.001 |
| gender:threatdistance | tokens collected | F(1,84)=2.161 | 0.145 |  | 0.435 |  | <0.001 |
| gender:threatdistance:threatlevel | tokens collected | F(1,84)=1.1 | 0.297 |  | 1.000 |  | <0.001 |
| gender:time | tokens collected | F(9.03,758.43)=6.18 | <0.001 | * | <0.001 | * | 0.018 |
| gender:time:threatlevel | tokens collected | F(10.76,903.57)=0.708 | 0.729 |  | 1.000 |  | 0.001 |
| gender:time:threatdistance | tokens collected | F(13.32,1118.94)=1.137 | 0.322 |  | 0.322 |  | 0.002 |
| gender:time:threatdistance:threatlevel | tokens collected | F(13.18,1107.26)=1.547 | 0.093 |  | 0.558 |  | 0.003 |
| time | tokens collected | F(9.03,758.43)=289.839 | <0.001 | * | <0.001 | * | 0.469 |
| time:threatlevel | tokens collected | F(10.76,903.57)=1.43 | 0.156 |  | 0.156 |  | 0.003 |
| time:threatdistance | tokens collected | F(13.32,1118.94)=3.417 | <0.001 | * | <0.001 | * | 0.006 |
| time:threatdistance:threatlevel | tokens collected | F(13.18,1107.26)=1.284 | 0.215 |  | 0.860 |  | 0.002 |
| yohimbine | tokens collected | F(1,84)=0.007 | 0.932 |  | 1.000 |  | <0.001 |
| yohimbine:threatlevel | tokens collected | F(1,84)=0.486 | 0.488 |  | 0.976 |  | <0.001 |
| yohimbine:threatdistance | tokens collected | F(1,84)=0.144 | 0.705 |  | 1.000 |  | <0.001 |
| yohimbine:threatdistance:threatlevel | tokens collected | F(1,84)=1.659 | 0.201 |  | 0.804 |  | <0.001 |
| yohimbine:gender | tokens collected | F(1,84)=1.946 | 0.167 |  | 1.000 |  | 0.005 |
| yohimbine:gender:threatlevel | tokens collected | F(1,84)=0 | 0.982 |  | 1.000 |  | <0.001 |
| yohimbine:gender:threatdistance | tokens collected | F(1,84)=6.815 | 0.011 | * | 0.066 |  | 0.001 |
| yohimbine:gender:threatdistance:threatlevel | tokens collected | F(1,84)=2.038 | 0.157 |  | 0.942 |  | <0.001 |
| yohimbine:gender:time | tokens collected | F(9.03,758.43)=0.758 | 0.656 |  | 1.000 |  | 0.002 |
| yohimbine:gender:time:threatlevel | tokens collected | F(10.76,903.57)=2.254 | 0.011 | * | 0.066 |  | 0.005 |
| yohimbine:gender:time:threatdistance | tokens collected | F(13.32,1118.94)=1.257 | 0.231 |  | 0.924 |  | 0.002 |
| yohimbine:gender:time:threatdistance:threatlevel | tokens collected | F(13.18,1107.26)=0.987 | 0.462 |  | 1.000 |  | 0.002 |
| yohimbine:time | tokens collected | F(9.03,758.43)=0.786 | 0.630 |  | 1.000 |  | 0.002 |
| yohimbine:time:threatlevel | tokens collected | F(10.76,903.57)=0.527 | 0.883 |  | 1.000 |  | 0.001 |
| yohimbine:time:threatdistance | tokens collected | F(13.32,1118.94)=0.623 | 0.840 |  | 1.000 |  | 0.001 |
| yohimbine:time:threatdistance:threatlevel | tokens collected | F(13.18,1107.26)=1.39 | 0.156 |  | 0.624 |  | 0.003 |
| hydrocortisone | distance to monster | F(1,84)=0.006 | 0.939 |  | 1.000 |  | <0.001 |
| hydrocortisone:threatlevel | distance to monster | F(1,84)=0.155 | 0.695 |  | 1.000 |  | <0.001 |
| hydrocortisone:threatdistance | distance to monster | F(1,84)=0.006 | 0.940 |  | 1.000 |  | <0.001 |
| hydrocortisone:threatdistance:threatlevel | distance to monster | F(1,84)=0.167 | 0.683 |  | 1.000 |  | <0.001 |
| hydrocortisone:gender | distance to monster | F(1,84)=0.068 | 0.795 |  | 1.000 |  | <0.001 |
| hydrocortisone:gender:threatlevel | distance to monster | F(1,84)=0.071 | 0.790 |  | 1.000 |  | <0.001 |
| hydrocortisone:gender:threatdistance | distance to monster | F(1,84)=0.656 | 0.420 |  | 1.000 |  | 0.001 |
| hydrocortisone:gender:threatdistance:threatlevel | distance to monster | F(1,84)=0.009 | 0.925 |  | 1.000 |  | <0.001 |
| hydrocortisone:gender:time | distance to monster | F(2.15,180.71)=0.076 | 0.937 |  | 1.000 |  | <0.001 |
| hydrocortisone:gender:time:threatlevel | distance to monster | F(3.51,295)=0.66 | 0.601 |  | 1.000 |  | <0.001 |
| hydrocortisone:gender:time:threatdistance | distance to monster | F(2.99,251.25)=0.359 | 0.782 |  | 1.000 |  | 0.001 |
| hydrocortisone:gender:time:threatdistance:threatlevel | distance to monster | F(4.07,341.87)=0.632 | 0.643 |  | 1.000 |  | <0.001 |
| hydrocortisone:time | distance to monster | F(2.15,180.71)=0.109 | 0.909 |  | 1.000 |  | <0.001 |
| hydrocortisone:time:threatlevel | distance to monster | F(3.51,295)=2.336 | 0.064 |  | 0.320 |  | 0.002 |
| hydrocortisone:time:threatdistance | distance to monster | F(2.99,251.25)=0.485 | 0.692 |  | 1.000 |  | 0.001 |
| hydrocortisone:time:threatdistance:threatlevel | distance to monster | F(4.07,341.87)=0.797 | 0.530 |  | 1.000 |  | <0.001 |
| hydrocortisone:yohimbine | distance to monster | F(1,84)=0.002 | 0.965 |  | 1.000 |  | <0.001 |
| hydrocortisone:yohimbine:threatlevel | distance to monster | F(1,84)=0.832 | 0.364 |  | 1.000 |  | <0.001 |
| hydrocortisone:yohimbine:threatdistance | distance to monster | F(1,84)=0.032 | 0.858 |  | 1.000 |  | <0.001 |
| hydrocortisone:yohimbine:threatdistance:threatlevel | distance to monster | F(1,84)=0.295 | 0.589 |  | 1.000 |  | <0.001 |
| hydrocortisone:yohimbine:gender | distance to monster | F(1,84)=0.704 | 0.404 |  | 1.000 |  | 0.003 |
| hydrocortisone:yohimbine:gender:threatlevel | distance to monster | F(1,84)=0.341 | 0.561 |  | 1.000 |  | <0.001 |
| hydrocortisone:yohimbine:gender:threatdistance | distance to monster | F(1,84)=1.636 | 0.204 |  | 0.715 |  | 0.002 |
| hydrocortisone:yohimbine:gender:threatdistance:threatlevel | distance to monster | F(1,84)=11.628 | 0.001 | * | 0.006 | * | 0.001 |
| hydrocortisone:yohimbine:gender:time | distance to monster | F(2.15,180.71)=1.008 | 0.372 |  | 1.000 |  | 0.003 |
| hydrocortisone:yohimbine:gender:time:threatlevel | distance to monster | F(3.51,295)=1.198 | 0.312 |  | 1.000 |  | 0.001 |
| hydrocortisone:yohimbine:gender:time:threatdistance | distance to monster | F(2.99,251.25)=1.057 | 0.368 |  | 1.000 |  | 0.002 |
| hydrocortisone:yohimbine:gender:time:threatdistance:threatlevel | distance to monster | F(4.07,341.87)=2.252 | 0.062 |  | 0.310 |  | 0.001 |
| hydrocortisone:yohimbine:time | distance to monster | F(2.15,180.71)=0.545 | 0.594 |  | 1.000 |  | 0.002 |
| hydrocortisone:yohimbine:time:threatlevel | distance to monster | F(3.51,295)=0.812 | 0.505 |  | 1.000 |  | 0.001 |
| hydrocortisone:yohimbine:time:threatdistance | distance to monster | F(2.99,251.25)=2.538 | 0.057 |  | 0.228 |  | 0.004 |
| hydrocortisone:yohimbine:time:threatdistance:threatlevel | distance to monster | F(4.07,341.87)=0.99 | 0.414 |  | 1.000 |  | 0.001 |
| threatlevel | distance to monster | F(1,84)=23.495 | <0.001 | * | <0.001 | * | 0.010 |
| threatdistance | distance to monster | F(1,84)=1292.128 | <0.001 | * | <0.001 | * | 0.590 |
| threatdistance:threatlevel | distance to monster | F(1,84)=7.783 | 0.007 | * | 0.036 | * | 0.001 |
| gender | distance to monster | F(1,84)=0.002 | 0.964 |  | 1.000 |  | <0.001 |
| gender:threatlevel | distance to monster | F(1,84)=0.088 | 0.767 |  | 1.000 |  | <0.001 |
| gender:threatdistance | distance to monster | F(1,84)=5.553 | 0.021 | * | 0.126 |  | 0.006 |
| gender:threatdistance:threatlevel | distance to monster | F(1,84)=0.017 | 0.898 |  | 1.000 |  | <0.001 |
| gender:time | distance to monster | F(2.15,180.71)=0.587 | 0.569 |  | 0.569 |  | 0.002 |
| gender:time:threatlevel | distance to monster | F(3.51,295)=1.666 | 0.166 |  | 0.996 |  | 0.001 |
| gender:time:threatdistance | distance to monster | F(2.99,251.25)=9.935 | <0.001 | * | <0.001 | * | 0.015 |
| gender:time:threatdistance:threatlevel | distance to monster | F(4.07,341.87)=0.32 | 0.868 |  | 1.000 |  | <0.001 |
| time | distance to monster | F(2.15,180.71)=654.125 | <0.001 | * | <0.001 | * | 0.667 |
| time:threatlevel | distance to monster | F(3.51,295)=6.17 | <0.001 | * | 0.001 | * | 0.004 |
| time:threatdistance | distance to monster | F(2.99,251.25)=2898.044 | <0.001 | * | <0.001 | * | 0.813 |
| time:threatdistance:threatlevel | distance to monster | F(4.07,341.87)=1.221 | 0.302 |  | 0.906 |  | 0.001 |
| yohimbine | distance to monster | F(1,84)=0.046 | 0.831 |  | 1.000 |  | <0.001 |
| yohimbine:threatlevel | distance to monster | F(1,84)=3.345 | 0.071 |  | 0.408 |  | 0.001 |
| yohimbine:threatdistance | distance to monster | F(1,84)=1.504 | 0.223 |  | 1.000 |  | 0.002 |
| yohimbine:threatdistance:threatlevel | distance to monster | F(1,84)=2.664 | 0.106 |  | 0.530 |  | <0.001 |
| yohimbine:gender | distance to monster | F(1,84)=0.049 | 0.825 |  | 1.000 |  | <0.001 |
| yohimbine:gender:threatlevel | distance to monster | F(1,84)=0.009 | 0.926 |  | 1.000 |  | <0.001 |
| yohimbine:gender:threatdistance | distance to monster | F(1,84)=0.015 | 0.901 |  | 1.000 |  | <0.001 |
| yohimbine:gender:threatdistance:threatlevel | distance to monster | F(1,84)=0.013 | 0.908 |  | 1.000 |  | <0.001 |
| yohimbine:gender:time | distance to monster | F(2.15,180.71)=0.423 | 0.671 |  | 1.000 |  | 0.001 |
| yohimbine:gender:time:threatlevel | distance to monster | F(3.51,295)=1.167 | 0.324 |  | 1.000 |  | 0.001 |
| yohimbine:gender:time:threatdistance | distance to monster | F(2.99,251.25)=0.312 | 0.816 |  | 1.000 |  | <0.001 |
| yohimbine:gender:time:threatdistance:threatlevel | distance to monster | F(4.07,341.87)=0.384 | 0.823 |  | 1.000 |  | <0.001 |
| yohimbine:time | distance to monster | F(2.15,180.71)=0.294 | 0.762 |  | 1.000 |  | 0.001 |
| yohimbine:time:threatlevel | distance to monster | F(3.51,295)=1.217 | 0.304 |  | 1.000 |  | 0.001 |
| yohimbine:time:threatdistance | distance to monster | F(2.99,251.25)=0.733 | 0.533 |  | 1.000 |  | 0.001 |
| yohimbine:time:threatdistance:threatlevel | distance to monster | F(4.07,341.87)=1.943 | 0.102 |  | 0.612 |  | 0.001 |
| hydrocortisone | distance to closest wall | F(1,84)=1.813 | 0.182 |  | 1.000 |  | 0.006 |
| hydrocortisone:threatlevel | distance to closest wall | F(1,84)=1.124 | 0.292 |  | 1.000 |  | <0.001 |
| hydrocortisone:threatdistance | distance to closest wall | F(1,84)=0.161 | 0.689 |  | 1.000 |  | <0.001 |
| hydrocortisone:threatdistance:threatlevel | distance to closest wall | F(1,84)=0.009 | 0.923 |  | 1.000 |  | <0.001 |
| hydrocortisone:gender | distance to closest wall | F(1,84)=0.028 | 0.867 |  | 1.000 |  | <0.001 |
| hydrocortisone:gender:threatlevel | distance to closest wall | F(1,84)=1.076 | 0.302 |  | 1.000 |  | <0.001 |
| hydrocortisone:gender:threatdistance | distance to closest wall | F(1,84)=0.603 | 0.439 |  | 1.000 |  | <0.001 |
| hydrocortisone:gender:threatdistance:threatlevel | distance to closest wall | F(1,84)=0.1 | 0.752 |  | 1.000 |  | <0.001 |
| hydrocortisone:gender:time | distance to closest wall | F(3.27,274.36)=0.173 | 0.927 |  | 1.000 |  | 0.001 |
| hydrocortisone:gender:time:threatlevel | distance to closest wall | F(5.47,459.21)=0.902 | 0.486 |  | 1.000 |  | 0.001 |
| hydrocortisone:gender:time:threatdistance | distance to closest wall | F(6,503.67)=0.601 | 0.729 |  | 1.000 |  | 0.001 |
| hydrocortisone:gender:time:threatdistance:threatlevel | distance to closest wall | F(7.16,601.72)=1.703 | 0.104 |  | 0.624 |  | 0.002 |
| hydrocortisone:time | distance to closest wall | F(3.27,274.36)=0.396 | 0.773 |  | 1.000 |  | 0.002 |
| hydrocortisone:time:threatlevel | distance to closest wall | F(5.47,459.21)=0.574 | 0.735 |  | 1.000 |  | 0.001 |
| hydrocortisone:time:threatdistance | distance to closest wall | F(6,503.67)=0.981 | 0.437 |  | 1.000 |  | 0.001 |
| hydrocortisone:time:threatdistance:threatlevel | distance to closest wall | F(7.16,601.72)=0.955 | 0.465 |  | 1.000 |  | 0.001 |
| hydrocortisone:yohimbine | distance to closest wall | F(1,84)=0.057 | 0.811 |  | 1.000 |  | <0.001 |
| hydrocortisone:yohimbine:threatlevel | distance to closest wall | F(1,84)=0.104 | 0.748 |  | 1.000 |  | <0.001 |
| hydrocortisone:yohimbine:threatdistance | distance to closest wall | F(1,84)=0.57 | 0.453 |  | 1.000 |  | <0.001 |
| hydrocortisone:yohimbine:threatdistance:threatlevel | distance to closest wall | F(1,84)=6.655 | 0.012 | * | 0.072 |  | 0.001 |
| hydrocortisone:yohimbine:gender | distance to closest wall | F(1,84)=0.713 | 0.401 |  | 1.000 |  | 0.002 |
| hydrocortisone:yohimbine:gender:threatlevel | distance to closest wall | F(1,84)=3.522 | 0.064 |  | 0.372 |  | 0.001 |
| hydrocortisone:yohimbine:gender:threatdistance | distance to closest wall | F(1,84)=0.16 | 0.690 |  | 0.715 |  | <0.001 |
| hydrocortisone:yohimbine:gender:threatdistance:threatlevel | distance to closest wall | F(1,84)=0.644 | 0.425 |  | 0.850 |  | <0.001 |
| hydrocortisone:yohimbine:gender:time | distance to closest wall | F(3.27,274.36)=1.269 | 0.285 |  | 1.000 |  | 0.005 |
| hydrocortisone:yohimbine:gender:time:threatlevel | distance to closest wall | F(5.47,459.21)=0.911 | 0.480 |  | 1.000 |  | 0.001 |
| hydrocortisone:yohimbine:gender:time:threatdistance | distance to closest wall | F(6,503.67)=1.097 | 0.363 |  | 1.000 |  | 0.001 |
| hydrocortisone:yohimbine:gender:time:threatdistance:threatlevel | distance to closest wall | F(7.16,601.72)=2.022 | 0.049 | * | 0.294 |  | 0.002 |
| hydrocortisone:yohimbine:time | distance to closest wall | F(3.27,274.36)=0.503 | 0.696 |  | 1.000 |  | 0.002 |
| hydrocortisone:yohimbine:time:threatlevel | distance to closest wall | F(5.47,459.21)=0.477 | 0.809 |  | 1.000 |  | 0.001 |
| hydrocortisone:yohimbine:time:threatdistance | distance to closest wall | F(6,503.67)=0.603 | 0.728 |  | 0.728 |  | 0.001 |
| hydrocortisone:yohimbine:time:threatdistance:threatlevel | distance to closest wall | F(7.16,601.72)=0.949 | 0.469 |  | 1.000 |  | 0.001 |
| threatlevel | distance to closest wall | F(1,84)=19.337 | <0.001 | * | <0.001 | * | 0.008 |
| threatdistance | distance to closest wall | F(1,84)=0.467 | 0.496 |  | 0.496 |  | <0.001 |
| threatdistance:threatlevel | distance to closest wall | F(1,84)=6.13 | 0.015 | * | 0.060 |  | 0.001 |
| gender | distance to closest wall | F(1,84)=3.839 | 0.053 |  | 0.265 |  | 0.013 |
| gender:threatlevel | distance to closest wall | F(1,84)=1.96 | 0.165 |  | 0.990 |  | 0.001 |
| gender:threatdistance | distance to closest wall | F(1,84)=0.871 | 0.353 |  | 0.435 |  | <0.001 |
| gender:threatdistance:threatlevel | distance to closest wall | F(1,84)=0.087 | 0.768 |  | 1.000 |  | <0.001 |
| gender:time | distance to closest wall | F(3.27,274.36)=4.696 | 0.002 | * | 0.006 | * | 0.019 |
| gender:time:threatlevel | distance to closest wall | F(5.47,459.21)=0.731 | 0.612 |  | 1.000 |  | 0.001 |
| gender:time:threatdistance | distance to closest wall | F(6,503.67)=2.124 | 0.049 | * | 0.098 |  | 0.003 |
| gender:time:threatdistance:threatlevel | distance to closest wall | F(7.16,601.72)=0.773 | 0.613 |  | 1.000 |  | 0.001 |
| time | distance to closest wall | F(3.27,274.36)=508.708 | <0.001 | * | <0.001 | * | 0.674 |
| time:threatlevel | distance to closest wall | F(5.47,459.21)=4.439 | <0.001 | * | 0.001 | * | 0.006 |
| time:threatdistance | distance to closest wall | F(6,503.67)=8.008 | <0.001 | * | <0.001 | * | 0.010 |
| time:threatdistance:threatlevel | distance to closest wall | F(7.16,601.72)=0.715 | 0.662 |  | 1.000 |  | 0.001 |
| yohimbine | distance to closest wall | F(1,84)=0.06 | 0.808 |  | 1.000 |  | <0.001 |
| yohimbine:threatlevel | distance to closest wall | F(1,84)=3.418 | 0.068 |  | 0.408 |  | 0.001 |
| yohimbine:threatdistance | distance to closest wall | F(1,84)=0.245 | 0.622 |  | 1.000 |  | <0.001 |
| yohimbine:threatdistance:threatlevel | distance to closest wall | F(1,84)=0.008 | 0.929 |  | 1.000 |  | <0.001 |
| yohimbine:gender | distance to closest wall | F(1,84)=0.611 | 0.437 |  | 1.000 |  | 0.002 |
| yohimbine:gender:threatlevel | distance to closest wall | F(1,84)=0.001 | 0.979 |  | 1.000 |  | <0.001 |
| yohimbine:gender:threatdistance | distance to closest wall | F(1,84)=5.699 | 0.019 | * | 0.095 |  | 0.001 |
| yohimbine:gender:threatdistance:threatlevel | distance to closest wall | F(1,84)=0.414 | 0.522 |  | 1.000 |  | <0.001 |
| yohimbine:gender:time | distance to closest wall | F(3.27,274.36)=0.447 | 0.736 |  | 1.000 |  | 0.002 |
| yohimbine:gender:time:threatlevel | distance to closest wall | F(5.47,459.21)=0.494 | 0.797 |  | 1.000 |  | 0.001 |
| yohimbine:gender:time:threatdistance | distance to closest wall | F(6,503.67)=1.645 | 0.133 |  | 0.665 |  | 0.002 |
| yohimbine:gender:time:threatdistance:threatlevel | distance to closest wall | F(7.16,601.72)=0.583 | 0.774 |  | 1.000 |  | 0.001 |
| yohimbine:time | distance to closest wall | F(3.27,274.36)=1.486 | 0.216 |  | 1.000 |  | 0.006 |
| yohimbine:time:threatlevel | distance to closest wall | F(5.47,459.21)=0.986 | 0.430 |  | 1.000 |  | 0.001 |
| yohimbine:time:threatdistance | distance to closest wall | F(6,503.67)=1.252 | 0.278 |  | 1.000 |  | 0.002 |
| yohimbine:time:threatdistance:threatlevel | distance to closest wall | F(7.16,601.72)=1.704 | 0.103 |  | 0.612 |  | 0.002 |
| hydrocortisone | presence in dangerous quadrant | F(1,84)=0.572 | 0.452 |  | 1.000 |  | 0.001 |
| hydrocortisone:threatlevel | presence in dangerous quadrant | F(1,84)=0.045 | 0.833 |  | 1.000 |  | <0.001 |
| hydrocortisone:threatdistance | presence in dangerous quadrant | F(1,84)=0.026 | 0.873 |  | 1.000 |  | <0.001 |
| hydrocortisone:threatdistance:threatlevel | presence in dangerous quadrant | F(1,84)=0.086 | 0.770 |  | 1.000 |  | <0.001 |
| hydrocortisone:gender | presence in dangerous quadrant | F(1,84)=0.877 | 0.352 |  | 1.000 |  | 0.002 |
| hydrocortisone:gender:threatlevel | presence in dangerous quadrant | F(1,84)=1.289 | 0.260 |  | 1.000 |  | <0.001 |
| hydrocortisone:gender:threatdistance | presence in dangerous quadrant | F(1,84)=0.409 | 0.524 |  | 1.000 |  | 0.001 |
| hydrocortisone:gender:threatdistance:threatlevel | presence in dangerous quadrant | F(1,84)=0.014 | 0.906 |  | 1.000 |  | <0.001 |
| hydrocortisone:gender:time | presence in dangerous quadrant | F(29,2436)=0.622 | 0.943 |  | 1.000 |  | 0.002 |
| hydrocortisone:gender:time:threatlevel | presence in dangerous quadrant | F(29,2436)=0.75 | 0.830 |  | 1.000 |  | 0.001 |
| hydrocortisone:gender:time:threatdistance | presence in dangerous quadrant | F(29,2436)=0.398 | 0.998 |  | 1.000 |  | 0.001 |
| hydrocortisone:gender:time:threatdistance:threatlevel | presence in dangerous quadrant | F(29,2436)=0.611 | 0.949 |  | 1.000 |  | 0.001 |
| hydrocortisone:time | presence in dangerous quadrant | F(29,2436)=0.347 | 1.000 |  | 1.000 |  | 0.001 |
| hydrocortisone:time:threatlevel | presence in dangerous quadrant | F(29,2436)=1.279 | 0.146 |  | 0.584 |  | 0.001 |
| hydrocortisone:time:threatdistance | presence in dangerous quadrant | F(29,2436)=0.272 | 1.000 |  | 1.000 |  | 0.001 |
| hydrocortisone:time:threatdistance:threatlevel | presence in dangerous quadrant | F(29,2436)=0.836 | 0.715 |  | 1.000 |  | 0.001 |
| hydrocortisone:yohimbine | presence in dangerous quadrant | F(1,84)=0.138 | 0.711 |  | 1.000 |  | <0.001 |
| hydrocortisone:yohimbine:threatlevel | presence in dangerous quadrant | F(1,84)=0.677 | 0.413 |  | 1.000 |  | <0.001 |
| hydrocortisone:yohimbine:threatdistance | presence in dangerous quadrant | F(1,84)=0.104 | 0.748 |  | 1.000 |  | <0.001 |
| hydrocortisone:yohimbine:threatdistance:threatlevel | presence in dangerous quadrant | F(1,84)=0.567 | 0.453 |  | 1.000 |  | <0.001 |
| hydrocortisone:yohimbine:gender | presence in dangerous quadrant | F(1,84)=0.401 | 0.528 |  | 1.000 |  | 0.001 |
| hydrocortisone:yohimbine:gender:threatlevel | presence in dangerous quadrant | F(1,84)=1.099 | 0.297 |  | 1.000 |  | <0.001 |
| hydrocortisone:yohimbine:gender:threatdistance | presence in dangerous quadrant | F(1,84)=1.479 | 0.227 |  | 0.715 |  | 0.002 |
| hydrocortisone:yohimbine:gender:threatdistance:threatlevel | presence in dangerous quadrant | F(1,84)=6.866 | 0.010 | * | 0.040 | * | 0.001 |
| hydrocortisone:yohimbine:gender:time | presence in dangerous quadrant | F(29,2436)=0.774 | 0.800 |  | 1.000 |  | 0.002 |
| hydrocortisone:yohimbine:gender:time:threatlevel | presence in dangerous quadrant | F(29,2436)=0.744 | 0.837 |  | 1.000 |  | 0.001 |
| hydrocortisone:yohimbine:gender:time:threatdistance | presence in dangerous quadrant | F(29,2436)=0.41 | 0.998 |  | 1.000 |  | 0.001 |
| hydrocortisone:yohimbine:gender:time:threatdistance:threatlevel | presence in dangerous quadrant | F(29,2436)=0.979 | 0.497 |  | 0.994 |  | 0.001 |
| hydrocortisone:yohimbine:time | presence in dangerous quadrant | F(29,2436)=0.952 | 0.538 |  | 1.000 |  | 0.003 |
| hydrocortisone:yohimbine:time:threatlevel | presence in dangerous quadrant | F(29,2436)=0.988 | 0.483 |  | 1.000 |  | 0.001 |
| hydrocortisone:yohimbine:time:threatdistance | presence in dangerous quadrant | F(29,2436)=1.526 | 0.036 | * | 0.180 |  | 0.004 |
| hydrocortisone:yohimbine:time:threatdistance:threatlevel | presence in dangerous quadrant | F(29,2436)=1.095 | 0.332 |  | 1.000 |  | 0.001 |
| threatlevel | presence in dangerous quadrant | F(1,84)=1.592 | 0.210 |  | 0.210 |  | <0.001 |
| threatdistance | presence in dangerous quadrant | F(1,84)=1590.282 | <0.001 | * | <0.001 | * | 0.708 |
| threatdistance:threatlevel | presence in dangerous quadrant | F(1,84)=0.422 | 0.518 |  | 0.518 |  | <0.001 |
| gender | presence in dangerous quadrant | F(1,84)=0.963 | 0.329 |  | 1.000 |  | 0.002 |
| gender:threatlevel | presence in dangerous quadrant | F(1,84)=0.837 | 0.363 |  | 1.000 |  | <0.001 |
| gender:threatdistance | presence in dangerous quadrant | F(1,84)=3.439 | 0.067 |  | 0.268 |  | 0.005 |
| gender:threatdistance:threatlevel | presence in dangerous quadrant | F(1,84)=1.617 | 0.207 |  | 1.000 |  | <0.001 |
| gender:time | presence in dangerous quadrant | F(29,2436)=2.581 | <0.001 | * | <0.001 | * | 0.007 |
| gender:time:threatlevel | presence in dangerous quadrant | F(29,2436)=0.957 | 0.532 |  | 1.000 |  | 0.001 |
| gender:time:threatdistance | presence in dangerous quadrant | F(29,2436)=5.888 | <0.001 | * | <0.001 | * | 0.016 |
| gender:time:threatdistance:threatlevel | presence in dangerous quadrant | F(29,2436)=0.933 | 0.569 |  | 1.000 |  | 0.001 |
| time | presence in dangerous quadrant | F(29,2436)=1650.631 | <0.001 | * | <0.001 | * | 0.827 |
| time:threatlevel | presence in dangerous quadrant | F(29,2436)=1.552 | 0.030 | * | 0.060 |  | 0.002 |
| time:threatdistance | presence in dangerous quadrant | F(29,2436)=2245.217 | <0.001 | * | <0.001 | * | 0.860 |
| time:threatdistance:threatlevel | presence in dangerous quadrant | F(29,2436)=1.377 | 0.087 |  | 0.435 |  | 0.001 |
| yohimbine | presence in dangerous quadrant | F(1,84)=0.499 | 0.482 |  | 1.000 |  | 0.001 |
| yohimbine:threatlevel | presence in dangerous quadrant | F(1,84)=2.638 | 0.108 |  | 0.432 |  | 0.001 |
| yohimbine:threatdistance | presence in dangerous quadrant | F(1,84)=2.72 | 0.103 |  | 0.618 |  | 0.004 |
| yohimbine:threatdistance:threatlevel | presence in dangerous quadrant | F(1,84)=4.338 | 0.040 | * | 0.240 |  | 0.001 |
| yohimbine:gender | presence in dangerous quadrant | F(1,84)=0.902 | 0.345 |  | 1.000 |  | 0.002 |
| yohimbine:gender:threatlevel | presence in dangerous quadrant | F(1,84)=1.047 | 0.309 |  | 1.000 |  | <0.001 |
| yohimbine:gender:threatdistance | presence in dangerous quadrant | F(1,84)=0.002 | 0.961 |  | 1.000 |  | <0.001 |
| yohimbine:gender:threatdistance:threatlevel | presence in dangerous quadrant | F(1,84)=0.139 | 0.710 |  | 1.000 |  | <0.001 |
| yohimbine:gender:time | presence in dangerous quadrant | F(29,2436)=1.84 | 0.004 | * | 0.024 | * | 0.005 |
| yohimbine:gender:time:threatlevel | presence in dangerous quadrant | F(29,2436)=1.043 | 0.403 |  | 1.000 |  | 0.001 |
| yohimbine:gender:time:threatdistance | presence in dangerous quadrant | F(29,2436)=2.169 | <0.001 | * | 0.002 | * | 0.006 |
| yohimbine:gender:time:threatdistance:threatlevel | presence in dangerous quadrant | F(29,2436)=0.329 | 1.000 |  | 1.000 |  | <0.001 |
| yohimbine:time | presence in dangerous quadrant | F(29,2436)=0.893 | 0.630 |  | 1.000 |  | 0.003 |
| yohimbine:time:threatlevel | presence in dangerous quadrant | F(29,2436)=0.355 | 0.999 |  | 1.000 |  | <0.001 |
| yohimbine:time:threatdistance | presence in dangerous quadrant | F(29,2436)=1.274 | 0.149 |  | 0.894 |  | 0.003 |
| yohimbine:time:threatdistance:threatlevel | presence in dangerous quadrant | F(29,2436)=0.779 | 0.795 |  | 1.000 |  | 0.001 |
| hydrocortisone | presence in safe place | F(1,84)=1.391 | 0.242 |  | 1.000 |  | 0.005 |
| hydrocortisone:threatlevel | presence in safe place | F(1,84)=0.467 | 0.496 |  | 1.000 |  | <0.001 |
| hydrocortisone:threatdistance | presence in safe place | F(1,84)=0.924 | 0.339 |  | 1.000 |  | <0.001 |
| hydrocortisone:threatdistance:threatlevel | presence in safe place | F(1,84)=0.225 | 0.637 |  | 1.000 |  | <0.001 |
| hydrocortisone:gender | presence in safe place | F(1,84)=0.001 | 0.975 |  | 1.000 |  | <0.001 |
| hydrocortisone:gender:threatlevel | presence in safe place | F(1,84)=0.406 | 0.526 |  | 1.000 |  | <0.001 |
| hydrocortisone:gender:threatdistance | presence in safe place | F(1,84)=1.519 | 0.221 |  | 1.000 |  | <0.001 |
| hydrocortisone:gender:threatdistance:threatlevel | presence in safe place | F(1,84)=0.672 | 0.415 |  | 1.000 |  | <0.001 |
| hydrocortisone:gender:time | presence in safe place | F(1.41,118.23)=0.123 | 0.811 |  | 1.000 |  | 0.001 |
| hydrocortisone:gender:time:threatlevel | presence in safe place | F(2.78,233.51)=0.595 | 0.606 |  | 1.000 |  | 0.001 |
| hydrocortisone:gender:time:threatdistance | presence in safe place | F(4.51,378.68)=0.766 | 0.562 |  | 1.000 |  | 0.001 |
| hydrocortisone:gender:time:threatdistance:threatlevel | presence in safe place | F(2.59,217.69)=1.604 | 0.196 |  | 0.980 |  | 0.001 |
| hydrocortisone:time | presence in safe place | F(1.41,118.23)=1.094 | 0.320 |  | 1.000 |  | 0.006 |
| hydrocortisone:time:threatlevel | presence in safe place | F(2.78,233.51)=0.624 | 0.588 |  | 1.000 |  | 0.001 |
| hydrocortisone:time:threatdistance | presence in safe place | F(4.51,378.68)=0.486 | 0.768 |  | 1.000 |  | 0.001 |
| hydrocortisone:time:threatdistance:threatlevel | presence in safe place | F(2.59,217.69)=0.317 | 0.784 |  | 1.000 |  | <0.001 |
| hydrocortisone:yohimbine | presence in safe place | F(1,84)=0.549 | 0.461 |  | 1.000 |  | 0.002 |
| hydrocortisone:yohimbine:threatlevel | presence in safe place | F(1,84)=0.604 | 0.439 |  | 1.000 |  | <0.001 |
| hydrocortisone:yohimbine:threatdistance | presence in safe place | F(1,84)=0.108 | 0.743 |  | 1.000 |  | <0.001 |
| hydrocortisone:yohimbine:threatdistance:threatlevel | presence in safe place | F(1,84)=1.354 | 0.248 |  | 1.000 |  | <0.001 |
| hydrocortisone:yohimbine:gender | presence in safe place | F(1,84)=0.006 | 0.937 |  | 1.000 |  | <0.001 |
| hydrocortisone:yohimbine:gender:threatlevel | presence in safe place | F(1,84)=0.955 | 0.331 |  | 1.000 |  | <0.001 |
| hydrocortisone:yohimbine:gender:threatdistance | presence in safe place | F(1,84)=4.34 | 0.040 | * | 0.240 |  | 0.001 |
| hydrocortisone:yohimbine:gender:threatdistance:threatlevel | presence in safe place | F(1,84)=0 | 0.983 |  | 0.983 |  | <0.001 |
| hydrocortisone:yohimbine:gender:time | presence in safe place | F(1.41,118.23)=0.164 | 0.771 |  | 1.000 |  | 0.001 |
| hydrocortisone:yohimbine:gender:time:threatlevel | presence in safe place | F(2.78,233.51)=1.339 | 0.263 |  | 1.000 |  | 0.001 |
| hydrocortisone:yohimbine:gender:time:threatdistance | presence in safe place | F(4.51,378.68)=1.78 | 0.124 |  | 0.496 |  | 0.002 |
| hydrocortisone:yohimbine:gender:time:threatdistance:threatlevel | presence in safe place | F(2.59,217.69)=0.347 | 0.762 |  | 0.994 |  | <0.001 |
| hydrocortisone:yohimbine:time | presence in safe place | F(1.41,118.23)=0.601 | 0.494 |  | 1.000 |  | 0.003 |
| hydrocortisone:yohimbine:time:threatlevel | presence in safe place | F(2.78,233.51)=0.594 | 0.607 |  | 1.000 |  | 0.001 |
| hydrocortisone:yohimbine:time:threatdistance | presence in safe place | F(4.51,378.68)=1.14 | 0.338 |  | 0.676 |  | 0.001 |
| hydrocortisone:yohimbine:time:threatdistance:threatlevel | presence in safe place | F(2.59,217.69)=0.434 | 0.700 |  | 1.000 |  | <0.001 |
| threatlevel | presence in safe place | F(1,84)=11.758 | 0.001 | * | 0.002 | * | 0.005 |
| threatdistance | presence in safe place | F(1,84)=477.415 | <0.001 | * | <0.001 | * | 0.087 |
| threatdistance:threatlevel | presence in safe place | F(1,84)=7.918 | 0.006 | * | 0.036 | * | 0.001 |
| gender | presence in safe place | F(1,84)=0.78 | 0.380 |  | 1.000 |  | 0.003 |
| gender:threatlevel | presence in safe place | F(1,84)=1.466 | 0.229 |  | 1.000 |  | 0.001 |
| gender:threatdistance | presence in safe place | F(1,84)=4.032 | 0.048 | * | 0.240 |  | 0.001 |
| gender:threatdistance:threatlevel | presence in safe place | F(1,84)=2 | 0.161 |  | 0.966 |  | <0.001 |
| gender:time | presence in safe place | F(1.41,118.23)=1.424 | 0.243 |  | 0.486 |  | 0.007 |
| gender:time:threatlevel | presence in safe place | F(2.78,233.51)=0.918 | 0.427 |  | 1.000 |  | 0.001 |
| gender:time:threatdistance | presence in safe place | F(4.51,378.68)=6.782 | <0.001 | * | <0.001 | * | 0.007 |
| gender:time:threatdistance:threatlevel | presence in safe place | F(2.59,217.69)=0.518 | 0.643 |  | 1.000 |  | <0.001 |
| time | presence in safe place | F(1.41,118.23)=162.313 | <0.001 | * | <0.001 | * | 0.452 |
| time:threatlevel | presence in safe place | F(2.78,233.51)=7.271 | <0.001 | * | 0.001 | * | 0.006 |
| time:threatdistance | presence in safe place | F(4.51,378.68)=737.547 | <0.001 | * | <0.001 | * | 0.450 |
| time:threatdistance:threatlevel | presence in safe place | F(2.59,217.69)=3.18 | 0.031 | * | 0.186 |  | 0.002 |
| yohimbine | presence in safe place | F(1,84)=0.041 | 0.839 |  | 1.000 |  | <0.001 |
| yohimbine:threatlevel | presence in safe place | F(1,84)=0 | 0.999 |  | 0.999 |  | <0.001 |
| yohimbine:threatdistance | presence in safe place | F(1,84)=1.508 | 0.223 |  | 1.000 |  | <0.001 |
| yohimbine:threatdistance:threatlevel | presence in safe place | F(1,84)=0.037 | 0.849 |  | 1.000 |  | <0.001 |
| yohimbine:gender | presence in safe place | F(1,84)=1.239 | 0.269 |  | 1.000 |  | 0.004 |
| yohimbine:gender:threatlevel | presence in safe place | F(1,84)=1.355 | 0.248 |  | 1.000 |  | 0.001 |
| yohimbine:gender:threatdistance | presence in safe place | F(1,84)=0.393 | 0.532 |  | 1.000 |  | <0.001 |
| yohimbine:gender:threatdistance:threatlevel | presence in safe place | F(1,84)=0.06 | 0.807 |  | 1.000 |  | <0.001 |
| yohimbine:gender:time | presence in safe place | F(1.41,118.23)=1.088 | 0.321 |  | 1.000 |  | 0.005 |
| yohimbine:gender:time:threatlevel | presence in safe place | F(2.78,233.51)=0.912 | 0.430 |  | 1.000 |  | 0.001 |
| yohimbine:gender:time:threatdistance | presence in safe place | F(4.51,378.68)=1.058 | 0.381 |  | 1.000 |  | 0.001 |
| yohimbine:gender:time:threatdistance:threatlevel | presence in safe place | F(2.59,217.69)=0.663 | 0.554 |  | 1.000 |  | <0.001 |
| yohimbine:time | presence in safe place | F(1.41,118.23)=0.279 | 0.678 |  | 1.000 |  | 0.001 |
| yohimbine:time:threatlevel | presence in safe place | F(2.78,233.51)=0.446 | 0.705 |  | 1.000 |  | <0.001 |
| yohimbine:time:threatdistance | presence in safe place | F(4.51,378.68)=0.87 | 0.493 |  | 1.000 |  | 0.001 |
| yohimbine:time:threatdistance:threatlevel | presence in safe place | F(2.59,217.69)=0.878 | 0.441 |  | 1.000 |  | 0.001 |
| hydrocortisone | presence in safe quadrant | F(1,84)=0.188 | 0.666 |  | 1.000 |  | 0.001 |
| hydrocortisone:threatlevel | presence in safe quadrant | F(1,84)=0.001 | 0.982 |  | 1.000 |  | <0.001 |
| hydrocortisone:threatdistance | presence in safe quadrant | F(1,84)=0.35 | 0.556 |  | 1.000 |  | <0.001 |
| hydrocortisone:threatdistance:threatlevel | presence in safe quadrant | F(1,84)=0.003 | 0.957 |  | 1.000 |  | <0.001 |
| hydrocortisone:gender | presence in safe quadrant | F(1,84)=0.002 | 0.969 |  | 1.000 |  | <0.001 |
| hydrocortisone:gender:threatlevel | presence in safe quadrant | F(1,84)=0.104 | 0.748 |  | 1.000 |  | <0.001 |
| hydrocortisone:gender:threatdistance | presence in safe quadrant | F(1,84)=0.447 | 0.506 |  | 1.000 |  | <0.001 |
| hydrocortisone:gender:threatdistance:threatlevel | presence in safe quadrant | F(1,84)=0.43 | 0.514 |  | 1.000 |  | <0.001 |
| hydrocortisone:gender:time | presence in safe quadrant | F(29,2436)=0.119 | 1.000 |  | 1.000 |  | <0.001 |
| hydrocortisone:gender:time:threatlevel | presence in safe quadrant | F(29,2436)=0.619 | 0.944 |  | 1.000 |  | 0.001 |
| hydrocortisone:gender:time:threatdistance | presence in safe quadrant | F(29,2436)=0.572 | 0.968 |  | 1.000 |  | 0.001 |
| hydrocortisone:gender:time:threatdistance:threatlevel | presence in safe quadrant | F(29,2436)=0.614 | 0.947 |  | 1.000 |  | 0.001 |
| hydrocortisone:time | presence in safe quadrant | F(29,2436)=0.296 | 1.000 |  | 1.000 |  | 0.001 |
| hydrocortisone:time:threatlevel | presence in safe quadrant | F(29,2436)=1.899 | 0.003 | * | 0.018 | * | 0.002 |
| hydrocortisone:time:threatdistance | presence in safe quadrant | F(29,2436)=1.207 | 0.206 |  | 1.000 |  | 0.002 |
| hydrocortisone:time:threatdistance:threatlevel | presence in safe quadrant | F(29,2436)=0.637 | 0.933 |  | 1.000 |  | 0.001 |
| hydrocortisone:yohimbine | presence in safe quadrant | F(1,84)=0.001 | 0.981 |  | 1.000 |  | <0.001 |
| hydrocortisone:yohimbine:threatlevel | presence in safe quadrant | F(1,84)=0.021 | 0.885 |  | 1.000 |  | <0.001 |
| hydrocortisone:yohimbine:threatdistance | presence in safe quadrant | F(1,84)=0.003 | 0.956 |  | 1.000 |  | <0.001 |
| hydrocortisone:yohimbine:threatdistance:threatlevel | presence in safe quadrant | F(1,84)=0.036 | 0.851 |  | 1.000 |  | <0.001 |
| hydrocortisone:yohimbine:gender | presence in safe quadrant | F(1,84)=0.35 | 0.556 |  | 1.000 |  | 0.001 |
| hydrocortisone:yohimbine:gender:threatlevel | presence in safe quadrant | F(1,84)=0.554 | 0.459 |  | 1.000 |  | <0.001 |
| hydrocortisone:yohimbine:gender:threatdistance | presence in safe quadrant | F(1,84)=2.113 | 0.150 |  | 0.715 |  | 0.001 |
| hydrocortisone:yohimbine:gender:threatdistance:threatlevel | presence in safe quadrant | F(1,84)=9.575 | 0.003 | * | 0.015 | * | 0.001 |
| hydrocortisone:yohimbine:gender:time | presence in safe quadrant | F(29,2436)=0.548 | 0.976 |  | 1.000 |  | 0.002 |
| hydrocortisone:yohimbine:gender:time:threatlevel | presence in safe quadrant | F(29,2436)=0.869 | 0.667 |  | 1.000 |  | 0.001 |
| hydrocortisone:yohimbine:gender:time:threatdistance | presence in safe quadrant | F(29,2436)=1.603 | 0.022 | * | 0.132 |  | 0.003 |
| hydrocortisone:yohimbine:gender:time:threatdistance:threatlevel | presence in safe quadrant | F(29,2436)=1.313 | 0.122 |  | 0.488 |  | 0.001 |
| hydrocortisone:yohimbine:time | presence in safe quadrant | F(29,2436)=1.744 | 0.008 | * | 0.048 | * | 0.006 |
| hydrocortisone:yohimbine:time:threatlevel | presence in safe quadrant | F(29,2436)=0.253 | 1.000 |  | 1.000 |  | <0.001 |
| hydrocortisone:yohimbine:time:threatdistance | presence in safe quadrant | F(29,2436)=2.503 | <0.001 | * | <0.001 | * | 0.004 |
| hydrocortisone:yohimbine:time:threatdistance:threatlevel | presence in safe quadrant | F(29,2436)=0.985 | 0.488 |  | 1.000 |  | 0.001 |
| threatlevel | presence in safe quadrant | F(1,84)=24.073 | <0.001 | * | <0.001 | * | 0.009 |
| threatdistance | presence in safe quadrant | F(1,84)=910.062 | <0.001 | * | <0.001 | * | 0.338 |
| threatdistance:threatlevel | presence in safe quadrant | F(1,84)=3.978 | 0.049 | * | 0.147 |  | <0.001 |
| gender | presence in safe quadrant | F(1,84)=0.038 | 0.845 |  | 1.000 |  | <0.001 |
| gender:threatlevel | presence in safe quadrant | F(1,84)=0.186 | 0.668 |  | 1.000 |  | <0.001 |
| gender:threatdistance | presence in safe quadrant | F(1,84)=1.645 | 0.203 |  | 0.435 |  | 0.001 |
| gender:threatdistance:threatlevel | presence in safe quadrant | F(1,84)=0.013 | 0.909 |  | 1.000 |  | <0.001 |
| gender:time | presence in safe quadrant | F(29,2436)=2.409 | <0.001 | * | <0.001 | * | 0.008 |
| gender:time:threatlevel | presence in safe quadrant | F(29,2436)=1.107 | 0.317 |  | 1.000 |  | 0.001 |
| gender:time:threatdistance | presence in safe quadrant | F(29,2436)=6.682 | <0.001 | * | <0.001 | * | 0.011 |
| gender:time:threatdistance:threatlevel | presence in safe quadrant | F(29,2436)=0.296 | 1.000 |  | 1.000 |  | <0.001 |
| time | presence in safe quadrant | F(29,2436)=396.83 | <0.001 | * | <0.001 | * | 0.558 |
| time:threatlevel | presence in safe quadrant | F(29,2436)=3.339 | <0.001 | * | <0.001 | * | 0.004 |
| time:threatdistance | presence in safe quadrant | F(29,2436)=882.829 | <0.001 | * | <0.001 | * | 0.596 |
| time:threatdistance:threatlevel | presence in safe quadrant | F(29,2436)=0.768 | 0.807 |  | 1.000 |  | 0.001 |
| yohimbine | presence in safe quadrant | F(1,84)=0.003 | 0.955 |  | 1.000 |  | <0.001 |
| yohimbine:threatlevel | presence in safe quadrant | F(1,84)=2.194 | 0.142 |  | 0.432 |  | 0.001 |
| yohimbine:threatdistance | presence in safe quadrant | F(1,84)=1.339 | 0.251 |  | 1.000 |  | 0.001 |
| yohimbine:threatdistance:threatlevel | presence in safe quadrant | F(1,84)=0.548 | 0.461 |  | 1.000 |  | <0.001 |
| yohimbine:gender | presence in safe quadrant | F(1,84)=0.094 | 0.760 |  | 1.000 |  | <0.001 |
| yohimbine:gender:threatlevel | presence in safe quadrant | F(1,84)=0.005 | 0.946 |  | 1.000 |  | <0.001 |
| yohimbine:gender:threatdistance | presence in safe quadrant | F(1,84)=0.002 | 0.961 |  | 1.000 |  | <0.001 |
| yohimbine:gender:threatdistance:threatlevel | presence in safe quadrant | F(1,84)=0.15 | 0.699 |  | 1.000 |  | <0.001 |
| yohimbine:gender:time | presence in safe quadrant | F(29,2436)=0.633 | 0.936 |  | 1.000 |  | 0.002 |
| yohimbine:gender:time:threatlevel | presence in safe quadrant | F(29,2436)=0.601 | 0.954 |  | 1.000 |  | 0.001 |
| yohimbine:gender:time:threatdistance | presence in safe quadrant | F(29,2436)=0.419 | 0.997 |  | 1.000 |  | 0.001 |
| yohimbine:gender:time:threatdistance:threatlevel | presence in safe quadrant | F(29,2436)=0.615 | 0.947 |  | 1.000 |  | 0.001 |
| yohimbine:time | presence in safe quadrant | F(29,2436)=0.476 | 0.992 |  | 1.000 |  | 0.002 |
| yohimbine:time:threatlevel | presence in safe quadrant | F(29,2436)=0.766 | 0.810 |  | 1.000 |  | 0.001 |
| yohimbine:time:threatdistance | presence in safe quadrant | F(29,2436)=1 | 0.466 |  | 1.000 |  | 0.002 |
| yohimbine:time:threatdistance:threatlevel | presence in safe quadrant | F(29,2436)=0.667 | 0.911 |  | 1.000 |  | 0.001 |

Note. The six over-trial parameters in ANOVAs with the between variables yohimbine, hydrocortisone, and gender and the within variables time, threat level (high/low) and initial threat distance (close/far). * in p<.05 and p.adj<.05 indicates significance for uncorrected and Bonferroni-Holm corrected p-values. Ges is the generalized eta square.

References

Bach DR, Guitart-Masip M, Packard PA, Miro J, Falip M, Fuentemilla L, Dolan RJ (2014) Human hippocampus arbitrates approach-avoidance conflict. Current biology : CB 24: 541-7.

Bach DR, Korn CW, Vunder J, Bantel A (2018) Effect of valproate and pregabalin on human anxiety-like behaviour in a randomised controlled trial. Translational psychiatry 8: 157.

Bach DR, Moutoussis M, Bowler A, Neuroscience in Psychiatry Network c, Dolan RJ (2020) Predictors of risky foraging behaviour in healthy young people. Nat Hum Behav 4: 832-843.

Beauducel A, Strobel A, Brocke B (2003) Psychometrische Eigenschaften und Normen einer deutschsprachigen Fassung der Sensation Seeking-Skalen, Form V. [Psychometric properties and norms of a German version of the Sensation Seeking Scales, Form V.]. Diagnostica 49: 61-72.

Fung BJ, Qi S, Hassabis D, Daw N, Mobbs D (2019) Slow escape decisions are swayed by trait anxiety. Nat Hum Behav.

Gao W, Stalder T, Kirschbaum C (2015) Quantitative analysis of estradiol and six other steroid hormones in human saliva using a high throughput liquid chromatography–tandem mass spectrometry assay. Talanta 143: 353-358.

Geyer M, Markou A (1995) Animal Models of Psychiatric Disorders. In: Bloom FE, Kupfer D (eds) Psychopharmacology: Fourth Generation of Progress. Raven Press, pp 787-798

Korn CW, Vunder J, Miró J, Fuentemilla L, Hurlemann R, Bach DR (2017) Amygdala Lesions Reduce Anxiety-like Behavior in a Human Benzodiazepine-Sensitive Approach-Avoidance Conflict Test. Biological psychiatry 82: 522-531.

Laux L, Glanzmann P, Schaffner P, Spielberger C (1981) Das State-Trait-Angstinventar (STAI) : theoretische Grundlagen und Handanweisung. Beltz Test GmbH, Weinheim

Rohleder N, Wolf JM, Maldonado EF, Kirschbaum C (2006) The psychosocial stress-induced increase in salivary alpha-amylase is independent of saliva flow rate. Psychophysiology 43: 645-52.

Shemesh Y, Chen A (2023) A paradigm shift in translational psychiatry through rodent neuroethology. Molecular Psychiatry.

Steyer R, Schwenkmezger P, Notz P, Eid M (1994) Testtheoretische Analysen des Mehrdimensionalen Befindlichkeitsfragebogen (MDBF). Diagnostica 40: 320-328.

Vogel S, Schwabe L (2019) Stress, aggression, and the balance of approach and avoidance. Psychoneuroendocrinology 103: 137-146.

Werner R, von Collani G (2014) Deutscher Aggressionsfragebogen. Zusammenstellung sozialwissenschaftlicher Items und Skalen.
